# Detecting Drawdowns Masked by Environmental Stresses with Water-Level Models

**DOI:** 10.1111/gwat.12042

**Published:** 2013-03-07

**Authors:** CA Garcia, KJ Halford, JM Fenelon

**Affiliations:** 2 U.S. Geological Survey2730 N. Deer Run Rd., Carson City, NV 89701.; 3 U.S. Geological Survey160 North Stephanie St., Henderson, NV 89704.

## Abstract

Detecting and quantifying small drawdown at observation wells distant from the pumping well greatly expands the characterized aquifer volume. However, this detection is often obscured by water level fluctuations such as barometric and tidal effects. A reliable analytical approach for distinguishing drawdown from nonpumping water-level fluctuations is presented and tested here. Drawdown is distinguished by analytically simulating all pumping and nonpumping water-level stresses simultaneously during the period of record. Pumping signals are generated with Theis models, where the pumping schedule is translated into water-level change with the Theis solution. This approach closely matched drawdowns simulated with a complex three-dimensional, hypothetical model and reasonably estimated drawdowns from an aquifer test conducted in a complex hydrogeologic system. Pumping-induced changes generated with a numerical model and analytical Theis model agreed (RMS as low as 0.007 m) in cases where pumping signals traveled more than 1 km across confining units and fault structures. Maximum drawdowns of about 0.05 m were analytically estimated from field investigations where environmental fluctuations approached 0.2 m during the analysis period.

## Introduction

The volume of aquifer system that can be characterized with aquifer tests is controlled by the distance at which drawdown can be detected. Drawdown detection typically is limited to distances of less than 1 km, because environmental water-level fluctuations frequently exceed the maximum displacement from pumping (Risser and Bird 2003).

Environmental fluctuations in measured water levels are described here as nonpumping (natural or anthropogenic) stresses on the aquifer system. These fluctuations include short-term, seasonal, and long-term stresses such as barometric pressure, tidal signals, natural and artificial recharge, and surface-water stage changes and diversions. Barometric pressure and tidal signals acting on the aquifer system can induce water-level changes of more than 0.3 m during periods of less than a few days (Fenelon 2000). Individual recharge events also can cause episodic water-level rises that exceed 0.5 m over a few days (O'Reilly 1998). Recharge likewise can induce long-term rising trends of more than 1 m/year that affect detection of small pumping signals (Fenelon 2000; Elliott and Fenelon 2010). Stage changes of a fully penetrating river can cause daily and event-based fluctuations in local groundwater levels (Criss and Criss 2011).

Environmental fluctuations from recharge responses, surface water stage changes, or any other external stress can be modeled explicitly by using water levels from background wells that are affected by these environmental stresses (Halford 2006; Criss and Criss 2011). A useful background well is one in which water levels are affected by tidal potential, imperfect barometric coupling between the atmosphere and water table, and all other stresses that affect water levels in observation wells excluding pumping. Although the need for background water levels to characterize environmental fluctuations has long been recognized (Stallman 1971), trends and corrections characterizing environmental fluctuations typically have been estimated qualitatively.

One method to reduce drawdown obscurity by environmental fluctuations is to interpret water-level changes from long-term (i.e., years) pumping of water-supply wells (Gonthier 2011; Harp and Vesselinov 2011). Long-term pumping can generate substantial drawdown over time, which will exceed environmental fluctuations during the period of analysis. Despite the utility of this method, using years of water-level changes from pumping of supply wells is an opportunistic approach that generally cannot be applied to aquifer tests.

Alternatively, environmental fluctuations have been distinguished from drawdown by modeling and directly removing barometric and tidal effects from measured water levels (Erskine 1991; Rasmussen and Crawford 1997; Toll and Rasmussen 2007). However, this approach does not remove environmental fluctuations caused by regional trends such as long-term recharge and is difficult to automate because all significant stresses that affect water levels other than pumping are not simulated explicitly.

The above approach was expanded upon by modeling and removing all nonpumping stresses from the water-level record (Halford 2006). Environmental fluctuations caused by barometric pressure and tidal signals, and regional trends captured in background water levels are modeled prior to pumping, projected forward during pumping and recovery periods, and removed from measured water levels. This approach requires antecedent monitoring periods that are more than three times longer than the pumping and recovery period in order to capture short and long-term environmental trends (Halford 2006). Considering recovery periods are often six times longer than pumping periods (Neville and van der Kamp 2012), this approach rapidly becomes unreliable where pumping periods exceed a week.

This paper describes and tests a method presented by Halford et al. (2012) for estimating observation-well drawdown response to aquifer-test stress where (1) environmental fluctuations mask the pumping signal, and (2) the period of record is limited. Drawdown is distinguished from environmental fluctuations by analytically simulating all water-level stresses (nonpumping and pumping) simultaneously during the period of record. Simultaneous modeling of environmental fluctuations and pumping signals overcomes the limitations of long-term interpretation and antecedent water-level monitoring. This approach draws on previous approaches where all environmental fluctuations are simulated and the pumping signal is modeled analytically using Theis (1935). Changes in pumping rates, multiple pumping wells, and lithologic variability are simulated by transforming multiple pumping schedules into water-level changes with Theis (1935). Transmissivity and storage coefficient are curve fitting parameters and are not interpreted as aquifer properties because the underlying assumptions of the Theis solution frequently are not met.

The described method was tested using “known” drawdowns generated from a hypothetical aquifer test that was simulated with a three-dimensional MODFLOW model. The utility of the approach also was demonstrated using a real aquifer test. The hypothetical model was conceptually based on the complex hydrogeology at the Nevada National Security Site (NNSS) at Pahute Mesa whereas the real aquifer test was conducted in a deep fractured volcanic rock aquifer at Pahute Mesa. The hypothetical model demonstrated that the drawdown estimation approach described here can closely match known drawdowns.

### Pahute Mesa Study Area

**Figure 1:**
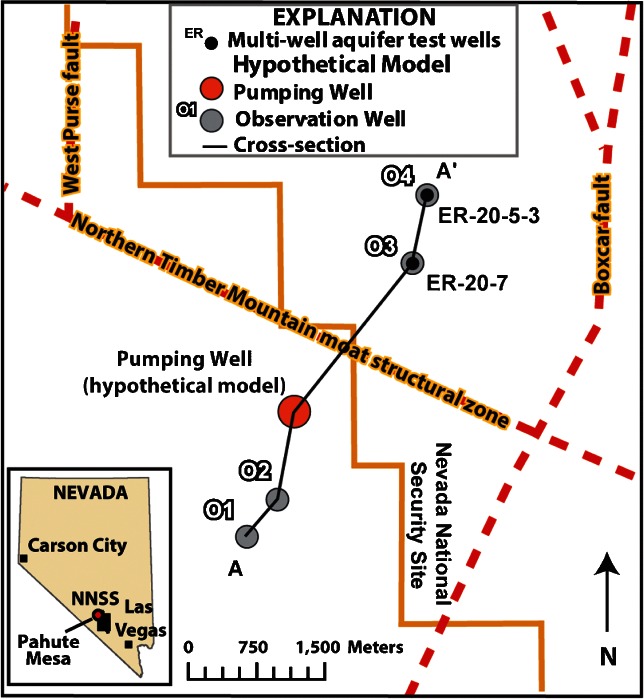
Location map showing Pahute Mesa wells and hypothetical pumping and observation locations.

Pahute Mesa is located in southern Nevada, within the NNSS (Figure 1). The aquifer system beneath Pahute Mesa comprises layered sequences of volcanic rocks that have been faulted into distinct structural blocks (Warren et al. 2000). Rhyolitic lavas or welded ash-flow tuffs such as in the Benham and Topopah Springs Aquifers, respectively, comprise aquifers. Bedded and nonwelded, zeolitized tuffs typically comprise confining units (Blankennagel and Weir 1973; Prothro and Drellack 1997; Bechtel Nevada 2002). More than a half dozen faults with offsets in excess of 200 m have been mapped previously in Pahute Mesa (McKee et al. 2001) and additional faults are mapped as well drilling continues (e.g., National Security Technologies, LLC 2010).

Distant drawdown detection from multiwell aquifer tests is necessary to quantify the hydraulic properties of the stratigraphic and structural features on Pahute Mesa so that radionuclide migration can be evaluated (Laczniak et al. 1996). The depth to water exceeds 600 m and sparsely distributed wells (generally more than 1 km apart) penetrate more than 1 km of a complex, volcanic rock-dominated, hydrogeologic system (Fenelon et al. 2010). Environmental water-level fluctuations are substantial beneath Pahute Mesa because of the thick unsaturated zone and high pneumatic and hydraulic diffusivity of the volcanic rocks.

## Methodology

The drawdown estimation approach was tested using hypothetical and real aquifer test applications. A three-dimensional MODFLOW model was used to generate “known” drawdowns in response to a hypothetical aquifer test. Environmental noise was then added to the known drawdowns in order to create a hypothetical water level where the pumping signal was masked. Known and unknown drawdowns from hypothetical and real aquifer tests, respectively, were detected using the drawdown estimation approach presented here by analytically simulating hypothetical and real measured water levels.

### Drawdown Estimation

Drawdowns in response to aquifer testing are estimated by analytically simulating all nonpumping and pumping water-level stresses simultaneously. These water-level models are calibrated to measured water levels during the entire period of aquifer-test data collection. Environmental fluctuations are modeled using input series of barometric pressure, tidal potential, and long-term recharge. Additional input series of water levels from background wells are included to capture environmental fluctuations from recharge responses or any other external nonpumping stress. Pumping signals are modeled by transforming pumping schedules into water-level responses using the Theis solution. Drawdown is computed as the summation of all Theis solutions and residual differences between measured and modeled water levels.

Measured water-level fluctuations are modeled analytically by summing multiple pumping and nonpumping stresses affecting the water-level record. All water-level stresses or components (WLC) are summed to compute a simulated water level (SWL) (Halford et al. 2012):


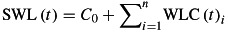
(1)

where *t* is time, *C*_0_ is a constant [L], *n* is the number of components, and WLC*_i_* is the *i*th water-level component in units of the modeled water level.

WLC include raw environmental fluctuations such as barometric pressure and computed components such as tide signals (Harrison 1971), moving averages of raw and computed nonpumping components, and pumping signals generated from the Theis solution (Halford et al. 2012). Nonpumping components are often transformed with multiple, moving averages to capture different signal frequencies. More than a half dozen water-level components frequently are created from a single input series because a broad range of averaging periods are more likely to simulate the environmental fluctuations.

A moving-average is applied to *i*th WLC at time, *t*, with:


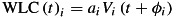
(2)

where *a*_*i*_ is the amplitude multiplier of the *i*th component in units of the modeled water level divided by units of the *i*th component, *φ*_*i*_ is the phase-shift of the *i*th component [T], and *V*_*i*_(*t* + *φ*_*i*_) is the value of the moving average of *i*th component at time *t* + *φ*_*i*_ in units of *i*th component. A WLC that was transformed with a moving average is adjusted by changing amplitude (*a*) and phase (*φ*) in Equation 2. Raw input series can be added as WLCs by assigning a moving average interval of 0 days. Summing multiple, moving averages of an input series is similar to the neural network approach (ASCE 2000).

**Figure 2:**
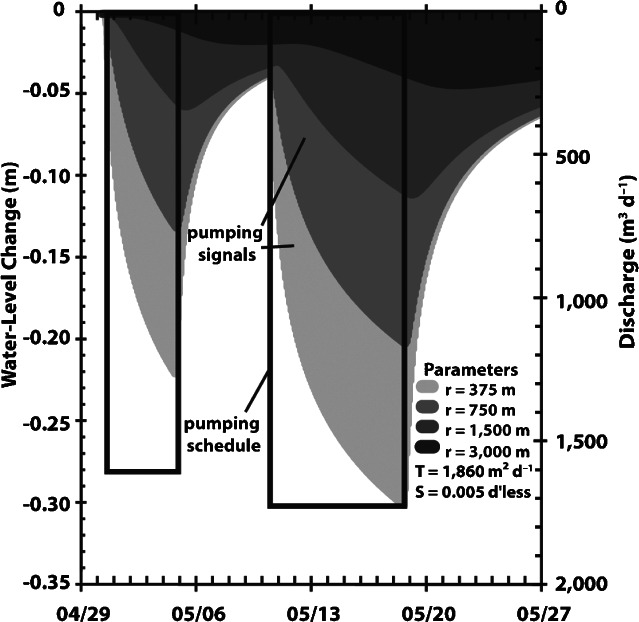
Water-level changes from a pumping schedule that were simulated with superimposed Theis solutions at multiple radial distances.

Pumping schedules are transformed into water-level responses with the Theis (1935) solution. Water-level change or drawdown, *s* [L], from pumping is simulated with:


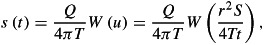
(3)

where *t* is the time since pumping commenced, *Q* is the pumping rate [L^3^/T], *T* is the transmissivity [L^2^/T], *W*(*u*) is the exponential integral solution, *u* is dimensionless time, *r* is the radial distance from the pumping well [L], and *S* is the storage coefficient [dimensionless]. Variable-rate pumping schedules (Figure 2) and multiple pumping wells can be simulated by superimposing multiple Theis solutions in time and space, respectively (Halford et al. 2012). Similar to the amplitude and phase shift of a moving average, a pumping signal generated with the Theis solution is adjusted by changing transmissivity (*T*) and storage coefficient (*S*) in Equation 3.

Transformation of step-wise pumping schedules into water-level changes at observation wells with superimposed Theis solutions (Equation 3) are discussed hereafter as Theis models. The term Theis model is introduced because superimposed Theis solutions typically are used as a transfer function to characterize the hydraulic properties of an aquifer system. In this approach, T and S are fitting parameters that do not necessarily represent hydraulic characteristics of the aquifer system. This is because homogeneity assumptions of the Theis solution are violated in hydrogeologically complex aquifer systems.

Water-level responses to pumping in hydrogeologically complex systems can be approximated well by applying multiple Theis models to a single pumping schedule. Pumping signals propagate through complex aquifer systems at different rates. Relatively faster and slower components of signals are approximated by the Theis models with correspondingly higher and lower hydraulic diffusivities. For example, fracture and matrix flow affect signal propagation in a dual-porosity system. Faster and slower signals, thus can be approximated by adjusting T and S parameters when generating pumping signals with the Theis solution. The summation of multiple pumping signals generated from a single pumping schedule can successfully simulate the water-level response from pumping in a complex non-Theis-like system.

Water-level models must be calibrated to reliably differentiate small pumping signals from environmental fluctuations. Differences between simulated and measured water levels are minimized with the parameter estimation tool PEST (Doherty 2008). Amplitude and phase or transmissivity and storage coefficient of each component series are adjusted. Sum-of-squares differences between simulated and measured water levels, or residuals, define the measurement objective function,


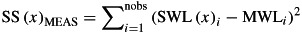
(4)

where *x* is the vector of parameters being estimated, nobs is the number of observations that are compared, SWL(*x*)*_i_* is the *i*th simulated water level, and MWL*_i_* is the *i*th measured water level. Although the sum-of-squares error serves as the measurement objective function, root-mean-square (RMS) error:


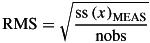
(5)

is reported in the analyses presented here because RMS is more comparable to water-level measurements.

Drawdown estimates are the summation of all calibrated pumping signals minus residuals. Residuals represent a composite of all water-level fluctuations that were not modeled with environmental- and pumping-component series. These fluctuations are predominantly random residuals during nonpumping periods but may contain components of the pumping signal during pumping periods that are not explained by the Theis solution.

Water levels are modeled and drawdowns were estimated with SeriesSEE, an Excel Add-In for viewing, cleaning, filtering, processing, and analyzing time-series data (Halford et al. 2012). Water-level records and components should be cleaned and filtered prior to simulating water levels. Water levels to be modeled, component time series, and period of analysis are defined interactively and viewed in Excel workbooks. Water levels are simulated with a FORTRAN program that is called from Excel. Parameter estimates, water-level components, simulated water levels, and water-level differences are imported automatically into the modeling workbook following parameter estimation by PEST. Parameters are estimated and water-level model results are evaluated iteratively until the user deems the fit to be adequate.

### Hypothetical Model

**Figure 3:**
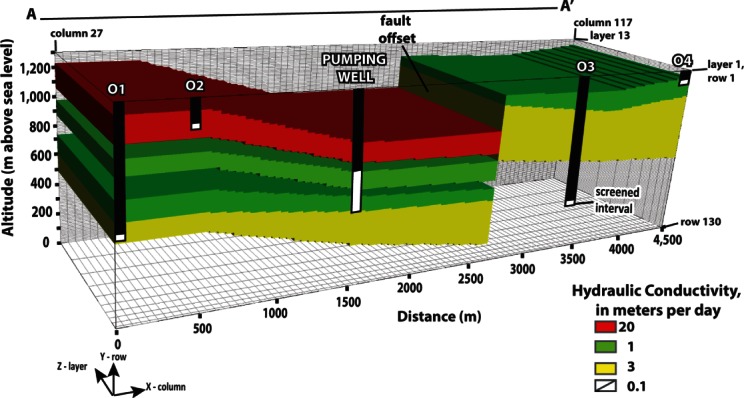
Cross section A-A′ (Figure 1), model grid, hydrogeologic framework, hydraulic-conductivity distribution, observation wells, and pumping well for hypothetical aquifer system. Colored areas represent aquifers and uncolored areas represent confining units.

The reliability of differentiating environmental fluctuations and pumping responses with the drawdown estimation approach was tested with a numerically simulated hypothetical aquifer test, for which drawdowns were known because they were simulated. This hypothetical system was designed with some degree of hydrogeologic complexity. The hydrogeologic framework was conceptualized as layered sequences of hydrostratigraphic units that were offset vertically more than 500 m across a major fault (Figure 3). The fault was simulated as an interface where hydrostratigraphic units were juxtaposed; the fault was not assigned unique hydraulic properties. This hypothetical sequence is similar to the vertical distribution of units that were mapped in cross sections beneath Pahute Mesa (National Security Technologies, LLC 2010).

The hypothetical aquifer system was simulated with a three-dimensional MODFLOW model (Harbaugh 2005). The three-dimensional model grid was rotated about the *x*-axis so the hydraulic-conductivity distribution coincided with model layer orientation (Figure 3). Therefore, with the layers conceptually flipped to the vertical position, the horizontal dimension was represented by columns and layers and the vertical dimension was represented by rows (Halford and Yobbi 2006). The model grid extended vertically from 0 to 1300 m above sea level, where the upper row of the model was the water table, and the 1300-m thickness was divided into uniform, 10-m thick rows. Temporal changes in the saturated thickness of the aquifer were not simulated because the maximum drawdown near the water table was small relative to the total thickness.

The model grid was divided into 144 columns, with the uniform part of the cross section (Figure 3) subdivided into 90, 50-m wide columns from 0 to 4500 m. The model domain extended laterally, beyond the cross section in Figure 3, and an additional 20,000 m in both directions where each additional column was 1.25 times wider than the previous column.

Symmetry was assumed about the cross section so only half of the hypothetical aquifer system was simulated explicitly. The cross section in Figure 3 was the plane of symmetry and was projected into the page. Layer 1 was half the 50-m thickness of layer 2 because of this plane of symmetry. Each additional layer was 1.25 times wider than the previous layer until the cross section projected more than 100 km into the page (only the first 13 layers are shown in Figure 3). All lateral boundaries were extended beyond 100 km so that simulated drawdowns were not affected by no-flow boundary conditions.

Hydraulic conductivity was distributed in layer 1 from 0 to 4500 m as shown in the cross section (Figure 3). The vertical distribution of hydraulic conductivity at 0 and 4500 m was extended left and right of the visible section to the model edges. Horizontal-to-vertical anisotropy of 1 was assigned. A uniform value of 0.02 was assigned for specific yield and 5 × 10^−6^/m was assigned for specific storage.

A hypothetical aquifer test was simulated and analyzed during a 5-month period that was divided into five stress periods. The pre-pumping, pumping, recovery, pumping, and recovery periods were 23, 10, 10, 10, and 97 days, respectively. Pumping rates were 1500 m^3^/d during the two 10-day pumping periods. The screened interval of the pumping well was simulated as a high-conductivity zone where water was removed from the uppermost cell and flow was apportioned across the interval within MODFLOW (Halford 2000).

**Figure 4:**
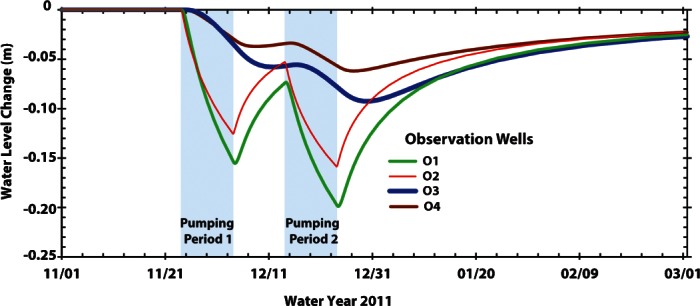
Known drawdown (numerically simulated pumping signals) observed at four locations (Figures 1 and 3) within the model domain.

Drawdowns simulated at four locations with the MODFLOW model, ranging between 1 and 3 km from the pumping well, were selected for further analysis (Figure 1). Water levels responded distinctly in observation wells O2 and O1 (Figure 4), which are 1 and 1.5 km, respectively, from the pumping well (Figure 3). Dampened responses occurred in observation wells O3 and O4 (Figure 4), which are 2.2 and 3 km, respectively, from the pumping well (Figure 3). A major fault that offsets the aquifers and confining units lies between the pumping well and observation wells O3 and O4 (Figure 1). These four simulated pumping responses will be referred to herein as known drawdowns.

**Figure 5:**
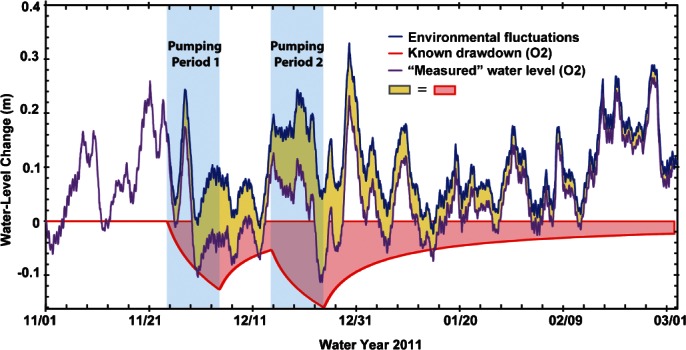
Known (numerically simulated) drawdown at model location O2 (Figure 1), environmental water-level fluctuations in Pahute Mesa background well UE-20n 1, and the hypothetical “measured” water level at location O2 (known drawdown + environmental fluctuations).

Hypothetical water levels at each observation well were created to emulate a real water-level response to pumping and test the drawdown estimation approach. Recognizing that known drawdowns represent pumping stresses only, hypothetical water levels were created using a combination of known drawdowns and measured environmental fluctuations. Measured water levels in background well UE-20n 1 (USGS National Water Information System [Bibr b31]) are assumed to be unaffected by real pumping beneath Pahute Mesa during the hypothetical model simulation period, and therefore, were assumed representative of environmental fluctuations only. Hypothetical water levels were created by adding measured water levels in background well UE-20n 1 to each known drawdown from November 1, 2010 to March 1, 2011 to imitate a real water-level response to pumping (Figure 5). The sum of water levels from well UE-20n 1 and known drawdowns are herein referred to as “measured” water levels that represent both pumping and nonpumping stresses.

## Results

### Hypothetical Model Application

**Figure 6:**
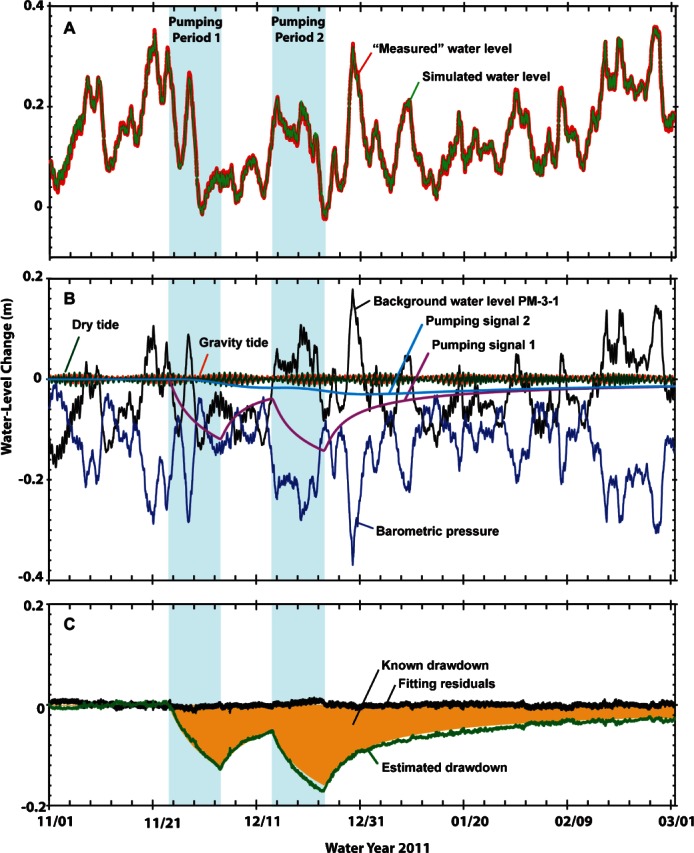
(A) “Measured” and analytically simulated water levels; (B) component time series; and (C) known (numerically simulated) drawdowns, analytically estimated drawdowns, and analytical fitting residuals in well O2 (Figure 1).

The reliability of differentiating drawdowns from environmental fluctuations with the drawdown estimation approach was tested by analytically simulating “measured” water levels where drawdowns were known (numerically simulated) (Figure 6A). Simulated water levels were created in the analytical model by combining water-level components representing environmental fluctuations and the pumping signal during the same period that the hypothetical aquifer test occurred.

Environmental water-level fluctuations were analytically simulated with component series including raw series and moving averages of barometric pressure and background water levels measured at Pahute Mesa background well PM-3-1 (located >12 km northwest of UE-20n 1; USGS National Water Information System, 2011), and computed dry- and gravity-earth tide components. Background well PM-3-1 penetrates similar volcanic units and is likely affected by similar environmental stresses as well UE-20n 1. Therefore it was assumed to sufficiently represent a combination of environmental fluctuations affecting the “measured” water levels, which were derived from well UE-20n 1.

Drawdown in well O2 was approximated with two Theis models in the analytical water-level model, because a single Theis model could not replicate the “known” pumping signal. Well O2 is screened in an aquifer that is separated by a confining unit from the aquifers that are penetrated by the pumping well (Figure 3). The need for two Theis models that conceptually approximate fast and slow aquifer responses can be anticipated where complex hydrogeology occurs. Differences between simulated and “measured” water levels were minimized by estimating 39 calibration coefficients during the period from November 1, 2010 to March 1, 2011 (Table 1; Figure 6).

**Table 1:** Component Time Series and Calibration Coefficients Used to Model Water Levels in Observation Well O2

		Calibration Coefficients		
			
Component	Time Series^1^,^2^	Amplitude (m)	Phase Shift (d)	Moving Averaging Interval^3^ (d)
Offset	—	0.01	—	—
Series	Background water level	0.59	−0.003	0
Series	Background water level	0.22	0.004	0.042
Series	Background water level	0.13	0.03	0.083
Series	Background water level	0.27	0.009	0.125
Series	Background water level	−0.15	−0.38	0.25
Series	Background water level	0.25	−0.40	0.5
Series	Background water level	−0.55	0.009	1
Series	Barometric pressure	−0.18	−0.01	0
Series	Barometric pressure	0.06	−0.06	0.042
Series	Barometric pressure	0.18	0.03	0.083
Series	Barometric pressure	−0.07	−0.28	0.125
Series	Barometric pressure	0.18	−0.18	0.25
Series	Barometric pressure	−0.01	−0.17	0.5
Series	Barometric pressure	−0.52	0.03	1
Series	Barometric pressure	0.029903	0.62	2
Tide	Computed gravity	0.00005	0.08	—
Tide	Computed dry	−0.00003	0.006	—
		**Transmissivity**^4^ **(m^2^/d)**	**StorageCoefficient**^4^ **(–)**	**Radius (m)**
		
Theis	Pumping signal	199	0.007	1,000
Theis	Pumping signal	176	0.10	1,000

*Note*: Component series include moving average transforms of background water levels and barometric pressure, earth tides (gravity and dry), and pumping signals generated with Theis models. Components were calibrated from November 1, 2010 to March 1, 2011.

1 Raw time series.

2 Background water levels and barometric pressure measured at Pahute Mesa well PM-3.

3 Units are the same as the raw time series.

4 Estimated values are fitting parameters that have no physical basis.

Physical significance should not be attributed to any of the calibration coefficients, especially T and S in the Theis models (Table 1). Known transmissivity that was specified in the numerical groundwater-flow model ranges between 3400 and 4400 m^2^/d left of the fault offset (Figure 3). Estimates of T in the two Theis models were less than 200 m^2^/d (Table 1). This considerable difference between calibration coefficients in Theis models and the known, complex hydraulic property distribution illustrates why hydraulic properties should not be interpreted directly from a water-level model.

Differences between analytically estimated and known (numerically simulated) drawdowns in well O2 averaged 0.005 m and maximum drawdowns differed by 6% (Figure 6C, Table 2). Analytically estimated drawdowns more than 1 month after pumping ceased were within 0.007 m, on average, of the known drawdowns.

**Table 2:** Analytically Simulated Water-Level Fit and Comparison Between Known and Estimated Drawdown at Four Locations

		Difference^2^ (m)		Maximum Drawdown (m)	
			
Model Location	RMS Error of Fit^1^ (m)	RMS Error of Prediction	Mean	Known	Estimated
O1	0.004	0.010	0.008	0.20	0.21
O2	0.004	0.007	0.005	0.16	0.17
O3	0.004	0.014	0.012	0.09	0.11
O4	0.004	0.008	0.006	0.06	0.07

1 RMS error describing analytical model calibration of simulated to “measured” water levels during the period of record, November 1, 2010 to March 1, 2011.

2 Difference between known (numerically simulated) and analytically estimated drawdown during pumping and recovery periods only, November 24, 2010 to March 1, 2011. Positive mean differences indicate underprediction.

**Figure 7:**
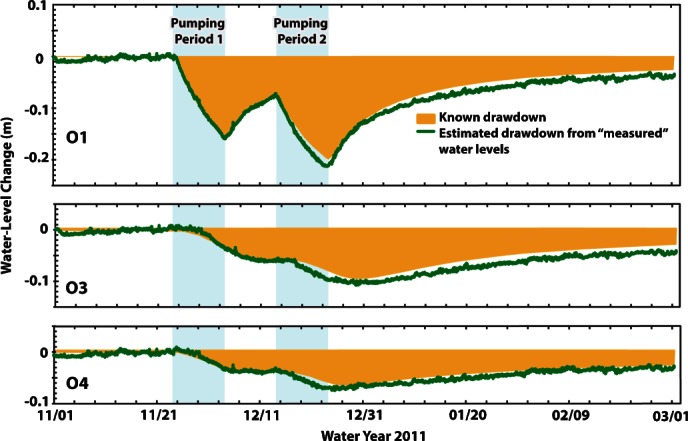
Known (numerically simulated) and analytically estimated drawdowns in observation wells O1, O3, and O4 (Figure 1). Drawdowns were estimated from “measured” water levels.

Drawdown also was estimated in wells O1, O3, and O4 with the analytical approach presented here (Figure 7, Table 2). The same environmental-fluctuation and pumping components were used in each approach as specified for well O2. Differences between analytically estimated and known drawdowns in wells O1, O3, and O4 averaged 0.008, 0.012, and 0.006 m, respectively. Root-mean-square fitting and prediction errors averaged 0.004 and 0.011 m, respectively, for wells O1, O3, and O4.

RMS errors of fit were consistent between observation wells and appear unrelated to the magnitude of drawdown (Table 2). Fitting errors were constant between observation locations (0.004 m) whereas maximum drawdown estimates varied by up to 0.14 m. This indicates that residual differences from the fit are driven by the measured environmental fluctuations rather than the drawdown magnitude or distance from the pumping well.

The prediction error (RMS error of prediction, Table 2) ranged between 1.75 and 3.5 times the modeled water-level fitting error (RMS error of fit, Table 2). On average, the prediction error shown here was about 2.5 times the fitting error. Similar results were found when known drawdowns were added to measured environmental fluctuations from two alternative background wells to compute “measured” water levels and estimate drawdown (data not shown). Considering results shown here and those determined with alternative well data, prediction errors were about 2.5 times the fitting errors on average and less than four times the fitting errors overall.

Nearly all differences between known and analytically estimated drawdowns result from noise in data sets, not use of the drawdown estimation approach. Known drawdowns that were sampled directly from the MODFLOW model results could be replicated with analytical Theis-generated pumping signals alone (no environmental fluctuations) with RMS prediction errors within 0.0005 m (not shown because signals appear identical). This is much less than the 0.007 m prediction error for well O2 between analytically estimated and known drawdowns where environmental fluctuations had been added to the known drawdowns (Table 2).

Maximum known and analytically estimated drawdowns agreed within 0.02 m in the four hypothetical wells. These small deviations are within the accuracy of the numerical solution of the hypothetical aquifer test. The hypothetical model, known drawdowns, measured barometric pressures, and measured water levels are available as supporting information.

### Aquifer Test Application

**Figure 8:**
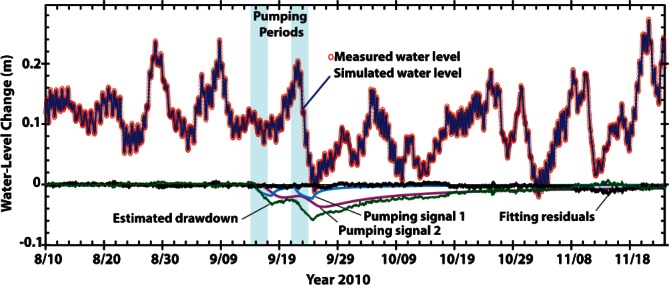
Measured and analytically simulated water levels, pumping signals, analytical fitting residuals, and analytically estimated drawdown in well ER-20-5-3 from pumping well ER-20-7 (Figure 1).

The utility of the drawdown estimation approach was demonstrated using a real aquifer test conducted in a deep fractured volcanic rock aquifer at Pahute Mesa (Halford et al. 2010). Drawdown in distant observation well ER-20-5-3 was estimated in response to pumping in well ER-20-7 (Figure 1). Well ER-20-7 produced about 17,500 m^3^ of water from the Topopah Spring aquifer during two pumping periods from September 14 to17 and 21 to 24, 2010 (Figure 8). Drawdown was estimated in observation well ER-20-5-3, which is screened in the Calico Hills zeolitic composite unit that is vertically offset by more than 100 m across a fault structure. Pumping and observation wells are 0.8 km apart (Figure 1) and penetrate different structural blocks.

The modeled fitting period was from August 10, 2010 to November 24, 2010. Multiple moving averages of barometric pressure and background water levels from well PM-3, and computed dry and gravity tides were used to model environmental fluctuations. The background well was nearly 8 km northwest of the pumping well, ER-20-7 (Halford et al. 2010). The pumping signal was analytically generated with two Theis models of the pumping schedule (Figure 8). Simulated water levels matched measured water levels with a RMS error of less than 0.003 m during the fitting period. Using fitting-to-prediction error comparisons from hypothetical model results, the drawdown prediction error is likely less than 0.01 m.

A maximum drawdown of about 0.05 m was estimated where environmental fluctuations exceeded 0.2 m over a day. Drawdown estimates in additional observation wells within 0.7 km of the pumping well and in wells >3 km away from the pumping well (Halford et al. 2010) corresponded with drawdown estimates in well ER-20-5-3 and ranged from <0.015 to 0.05 m. Consistent distance-drawdown responses in all wells within a 3 km radius of the pumping well indicate that the ER-20-7 drawdown estimates are plausible.

## Conclusions

The analytical drawdown estimation approach described here is shown to be reliable for estimating distant observation well response to aquifer-test stress where (1) environmental noise masks the pumping signal, and (2) the antecedent monitoring period is limited. With this approach, environmental water-level fluctuations caused by natural stress on the aquifer system, and the pumping signal from aquifer testing are analytically simulated simultaneously and distinguished from one another during pre-pumping, pumping, and recovery periods in order to estimate drawdown. Environmental fluctuations primarily are simulated with barometric pressure, background water-level, and computed dry- and gravity-tide components. Pumping signals are generated with Theis models, where step-wise pumping records of discharge are transformed into water-level changes using multiple superimposed Theis solutions. This approach closely approximated drawdowns that were numerically simulated with a complex three-dimensional, hypothetical model and reasonably estimated drawdowns from an aquifer test conducted in a complex hydrogeologic system.

Drawdown responses to pumping that were numerically simulated with a complex three-dimensional MODFLOW model were closely approximated using the analytical drawdown estimation approach. Known (numerically simulated) drawdowns were analytically estimated within volcanic aquifers penetrating the same unit as the pumping well, in aquifers separated from the pumping well by tuff confining units, and in aquifers and confining units separated and vertically displaced from the pumping well by a major fault. Known drawdowns were added to the water-level record measured in a real background well, which represents environmental fluctuations only, in order to create hypothetical “measured” water-level records.

Differences between known and analytically estimated drawdowns result almost exclusively from noise in the measured time series. Pumping signals generated with Theis models could match hypothetical model output with RMS errors of prediction (differences between known and estimated drawdowns) of less than 0.0005 m. These RMS errors of prediction increased by more than 10 times when matching hypothetical “measured” water-level records where known drawdowns were obscured by environmental fluctuations. RMS errors of prediction for noiseless drawdowns were insignificant relative to drawdowns estimated from hypothetical “measured” water-level records.

Fitting errors between measured and analytically simulated water levels approximate the error associated with estimating drawdown during an aquifer test. RMS errors of prediction averaged less than three times the fitting errors between simulated and measured water levels. This facilitates estimating the overall measurement error, which is necessary for interpreting aquifer-test results with a highly parameterized, groundwater-flow model.

## Supporting Information

**Appendix S1.** File structure for hypothetical model (Hsieh and Winston 2002).

**Figure S1.** MODFLOW (MF) and water-level modeling (WLM) subfolders for the hypothetical model.

**Figure S2.** DFLOW, Batch, and Array files for the hypothetical model.

**Figure S3.** Hydraulic conductivity arrays for the hypothetical model.

**Figure S4.** Hydrographs of simulated “known” draw-downs for several observation points from the hypothetical model. These hydrographs were created from observation well results.

**Figure S5.** Water-level model files and input time-series data including continuous water levels in background wells, known drawdowns, barometric pressure, and pumping schedules for analysis of hypothetical model results.

**Figure S6.** Additional water-level models for hypothetical model results.

**Figure S7.** Water-level model files and input time-series for analysis of the Pahute Mesa (PM) ER-20-5-3 drawdown response to pumping well ER-20-7 in the Pahute_Mesa_Ex.zip file.
