# Supplementary material for: Detecting Drawdowns Masked by Environmental Stresses with Water-Level Models
Source: Ground Water. 2013 Mar 7;51(3):322–32. doi: 10.1111/gwat.12042 (PMC3675638; doi:10.1111/gwat.12042)
Supplement: Supplementary file 1 [file gwat0051-0322-SD1.pdf]

Contents

File Structure for Hypothetical Model ….…………………………………………………………………………..3

File Structure for Water Level Modeling in Pahute Mesa ……………………...…………..……………………...10

Figures

Figure 1. Root directory and files for Hypothetical Model and Water Level Modeling at Pahute Mesa (PM) …....4

Figure 2. MODFLOW (MF) and water-level modeling (WLM) subfolders for the hypothetical model in the Hypothetical_Model.zip file………………...…………..……………… ………………………………………5

Figure 3. DFLOW, Batch, and Array files for the hypothetical model in the Hypothetical_Model.zip\MF subfolder……………..…………..…………..…………………………………………………….…………6

Figure 4. Hydraulic conductivity arrays for the hypothetical model in the Hypothetical_Model.zip\MF\Arrays subfolder……………………………………………………………………….……………………...…7

Figure 5. Hydrographs of simulated "known" drawdowns for several observation points from the hypothetical model in the Hypothetical_Model.zip\MF\Hydros subfolder. These hydrographs were created from observation well results in the Pcomp_HypoModel.WLsimulated.txt file located in the Hypothetical_Model.zip\MF subfolder………………………. ……………………………………………………....……………………….8

Figure 6. Water-level model files and input time-series data including continuous water levels in background wells, known drawdowns, barometric pressure, and pumping schedules for analysis of hypothetical model results in the Hypothetical_Model.zip\WLM ……………………..……..……………………………………...…9

Figure 7. Additional water-level models for hypothetical model results in the Hypothetical_Model.zip\WLM\AdditionalExamples subfolder ……………………..……………………..…….10

Figure 8. Water-level model files and input time-series for analysis of the Pahute Mesa (PM) ER-20-5-3 drawdown response to pumping well ER-20-7 in the Pahute_Mesa_Ex.zip file………………..……………………....…12

File Structure for Hypothetical Model

The hypothetical three-dimensional model is located in the *HypotheticalModel.zip* file (Fig. 1) and is comprised of MODFLOW model (MF) and water-level modeling (WLM) sub-folders (Fig. 1). The MODFLOW, batch, and array files are in the *MF* subdirectory. The MODFLOW files include input and output files, an excel file with model discretization, and model viewer files for hydraulic conductivity (k) and head distributions. Hypothetical model observation wells O1, O2, O3, and O4 presented in the manuscript are synonymous with SupportingInformation observation wells CHZ-x0, BA-EC-6, CFC-20-7, and TSA-20-5-1, respectively. Model viewer can be downloaded at *http://water.usgs.gov/nrp/gwsoftware/modelviewer/ModelViewer.html*. The *MF* folder has two subdirectories: Arrays and Hydros (Fig. 2). The *Arrays* subdirectory is comprised of hydraulic conductivity arrays used in the MODFLOW model (Fig. 3). Simulated drawdown hydrographs for observation points output from MODFLOW are in the *Hydros* subdirectory (Fig. 4).

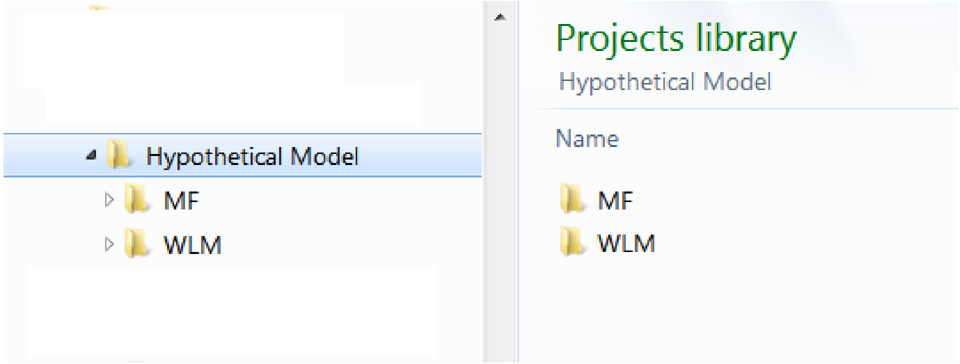

Figure 1. MODFLOW (MF) and water-level modeling (WLM) subfolders for the hypothetical model in the Hypothetical_Model.zip file.

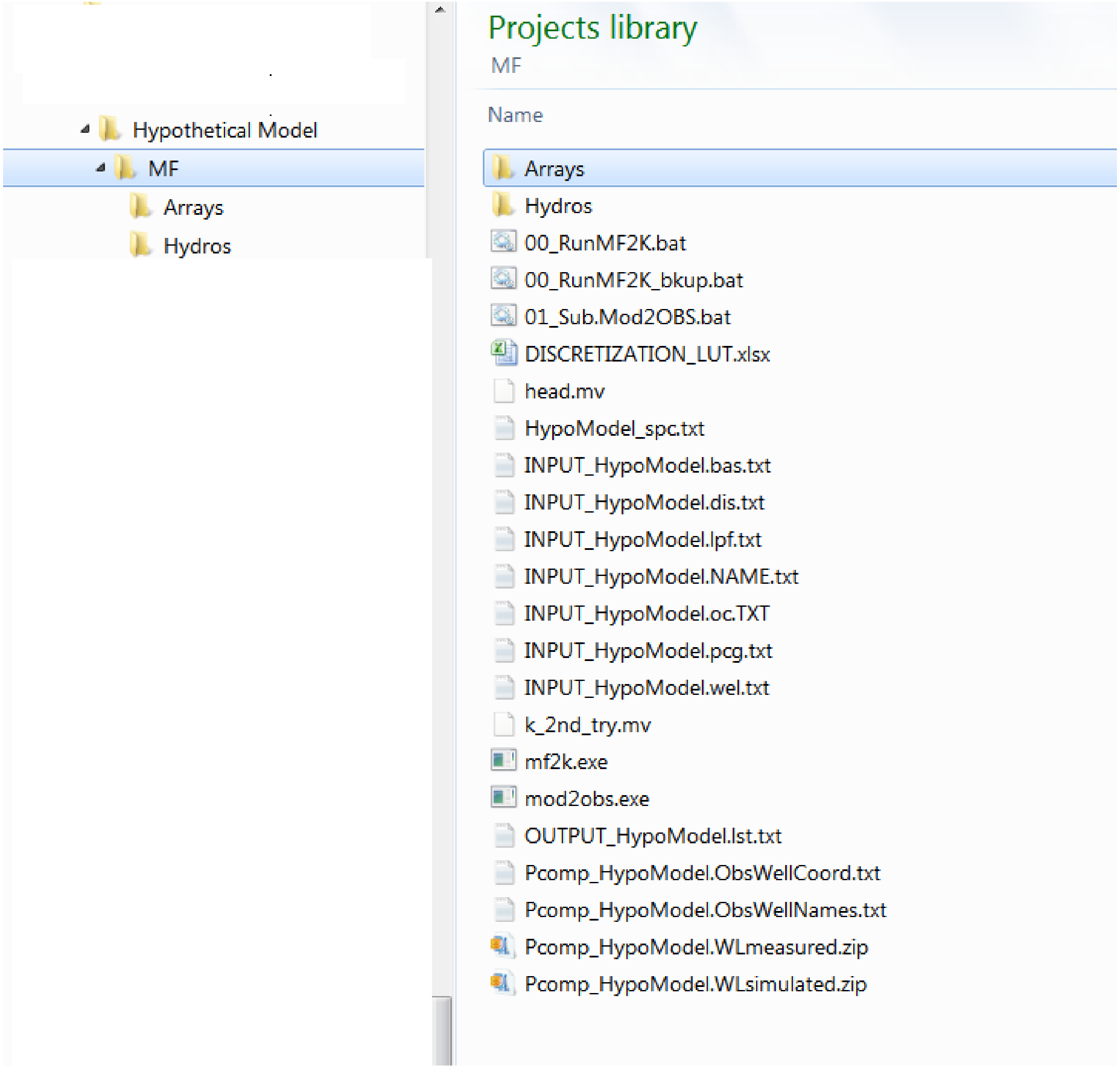

Figure 2. MODFLOW, Batch, and Array files for the hypothetical model in the Hypothetical_Model.zip\MF subfolder.

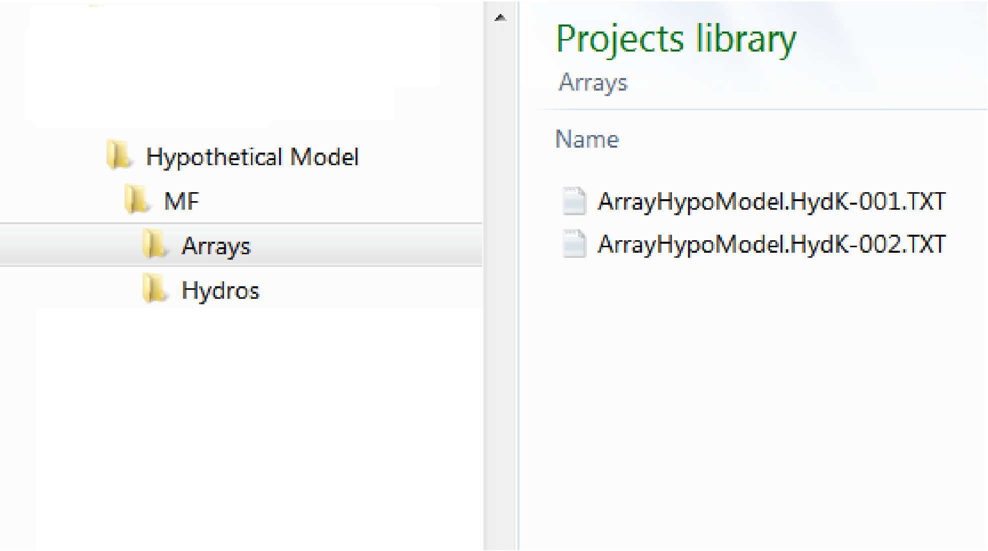

Figure 3. Hydraulic conductivity arrays for the hypothetical model in the Hypothetical_Model.zip\MF\Arrays subfolder.

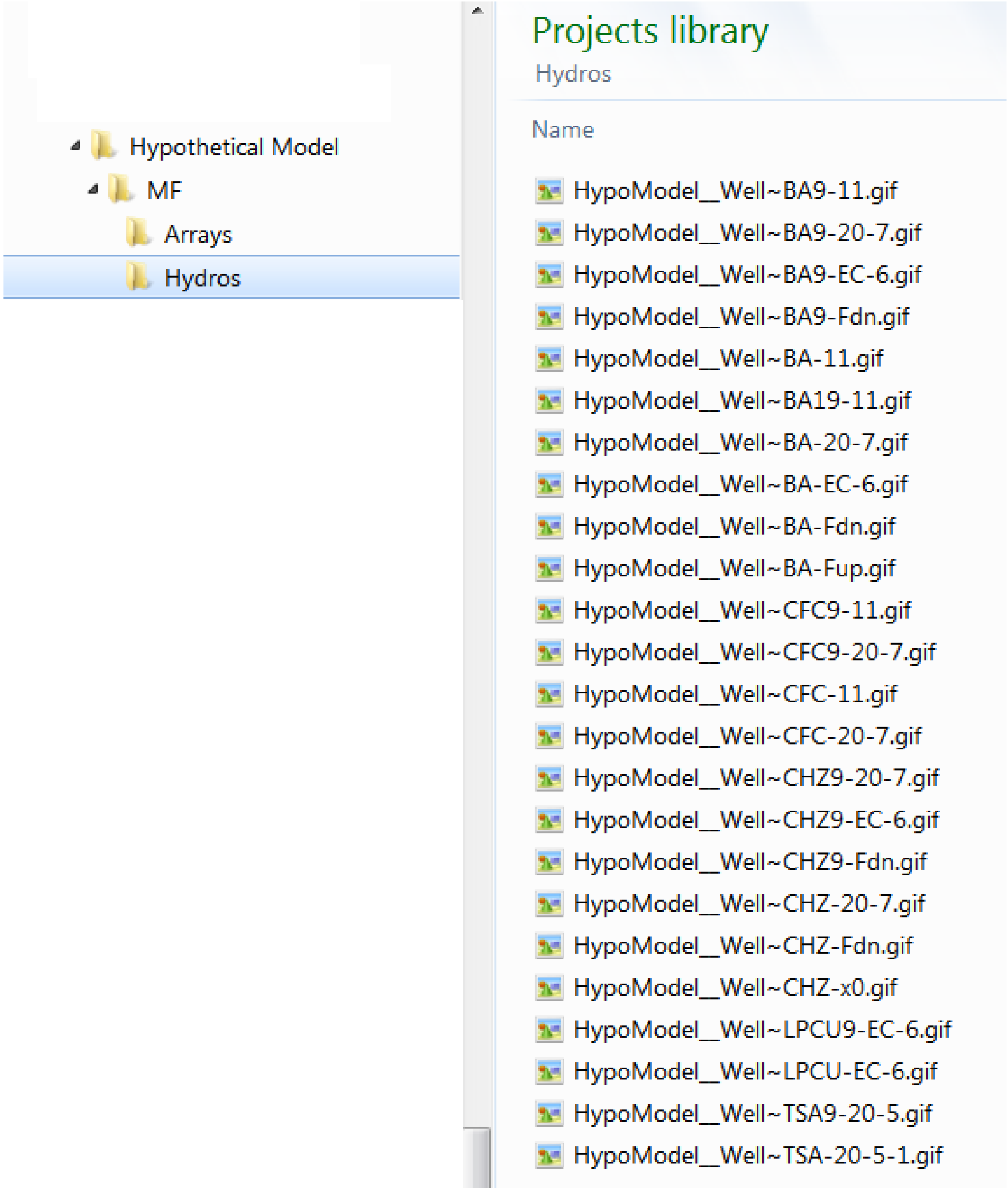

Figure 4. Hydrographs of simulated "known" drawdowns for several observation points from the hypothetical model in the Hypothetical_Model.zip\MF\Hydros subfolder. These hydrographs were created from observation well results in the Pcomp_HypoModel.WLsimulated.txt file located in the Hypothetical_Model.zip\MF subfolder. Observation wells CHZ-x0, BA-EC-6, CFC-20-7, and TSA-20-5-1 are synonymous with manuscript observation wells O1, O2, O3, and O4, respectively.

Simulated drawdown from the hypothetical model (equivalent to the “known” drawdown in the manuscript) was estimated using water-level models. Water-level model files for the four wells in the manuscript are located in the *WLM* subfolder below *Hypothetical_Model.zip* (Fig. 5). This directory also contains the input time series data such as “known” drawdown, background water levels, barometric pressure, and contains additional water-level-models where alternative background water-levels were modeled in subdirectory *AdditionalExamples* (Fig. 6).

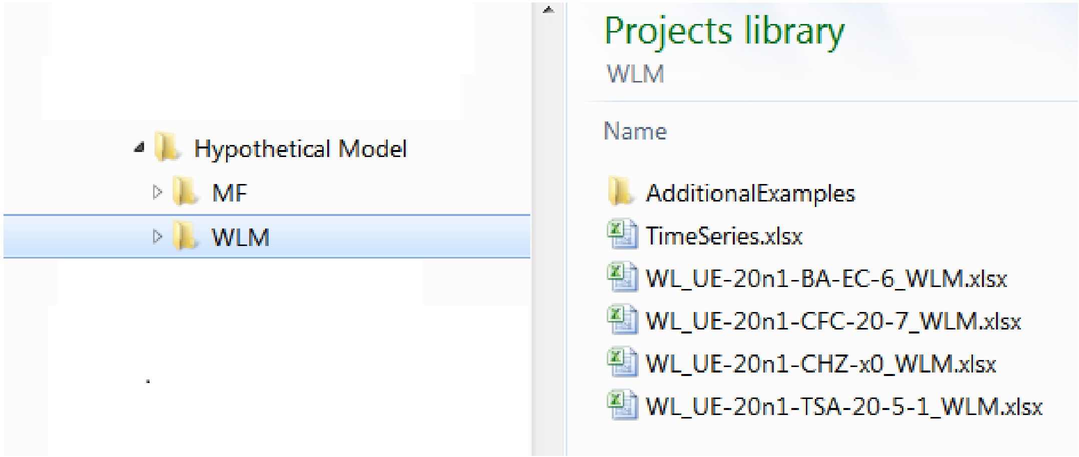

Figure 5. Water-level model files and input time-series data including continuous water levels in background wells, known drawdowns, barometric pressure, and pumping schedules for analysis of hypothetical model results in the Hypothetical_Model.zip\WLM subfolder. Observation wells CHZ-x0, BA-EC-6, CFC-20-7, and TSA-20-5-1 are synonymous with manuscript observation wells O1, O2, O3, and O4, respectively.

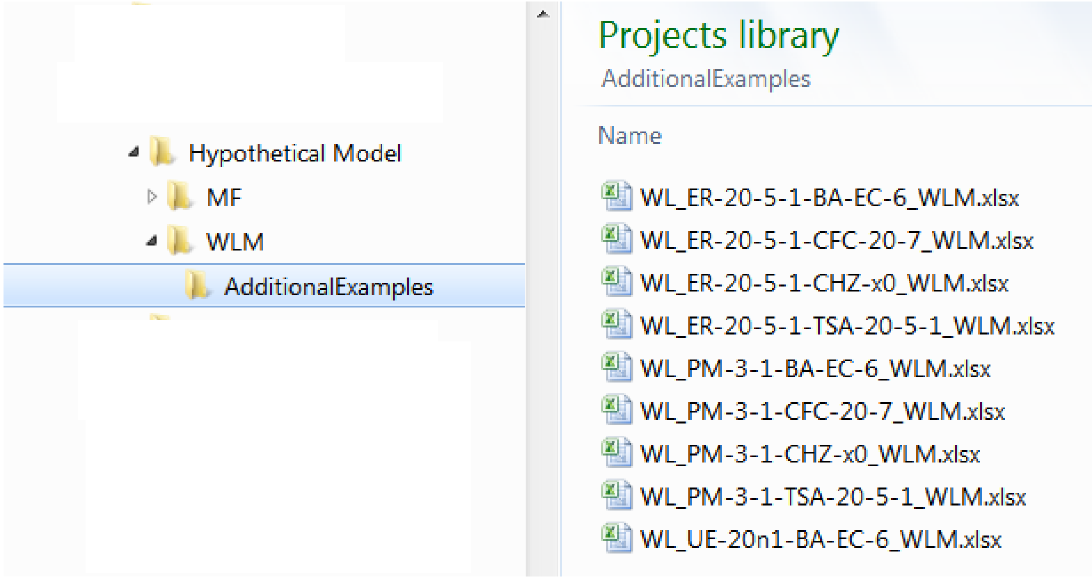

Figure 6. Additional water-level models for hypothetical model results in the Hypothetical_Model.zip\WLM\AdditionalExamples subfolder. Observation wells CHZ-x0, BA-EC-6, CFC-20-7, and TSA-20-5-1 are synonymous with manuscript observation wells O1, O2, O3, and O4, respectively.

File Structure for Water Level Modeling in Pahute Mesa

Water Level Models for Pahute Mesa multi-well aquifer test are located in the *Pahute_Mesa_Ex.zip* file. The Pahute Mesa directory shows drawdown response in well ER-20-5-3 to pumping well ER-20-7 (Figure 7). This folder contains water-level models using the analytical drawdown estimation approach, and time-series data such as the pumping schedule, background water levels, and barometric pressure used to model environmental fluctuations. Theis models, earth-tides, and fitting errors are located within water-level model workbooks.

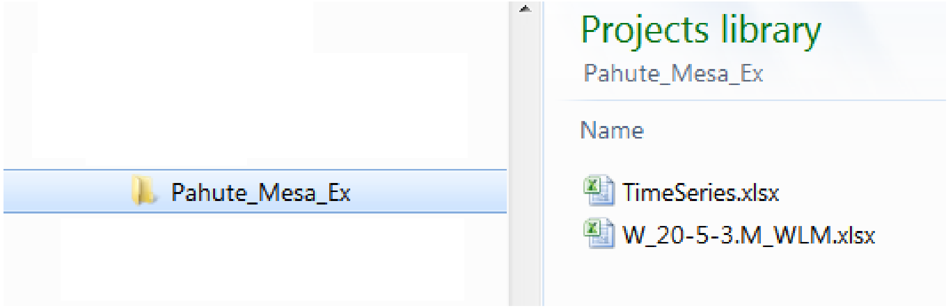

Figure 7. Water-level model files and input time-series for analysis of the Pahute Mesa (PM) ER-20-5-3 drawdown response to pumping well ER-20-7 in the Pahute_Mesa_Ex.zip file.
