# Supplementary figures and images for: Detecting Drawdowns Masked by Environmental Stresses with Water-Level Models
Source: Ground Water. 2013 Mar 7;51(3):322–32. doi: 10.1111/gwat.12042 (PMC3675638; doi:10.1111/gwat.12042)

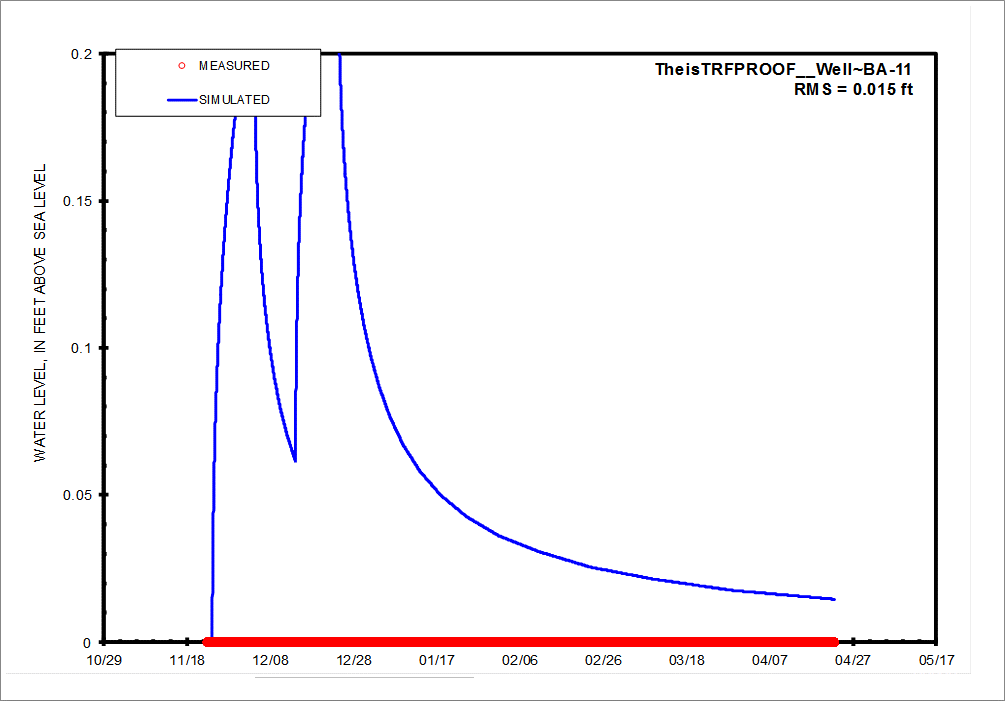

Supplement: Supplementary file 2 [file gwat0051-0322-SD2.zip › HypotheticalModel/MF/Hydros/HypoModel__Well~BA-11.gif]

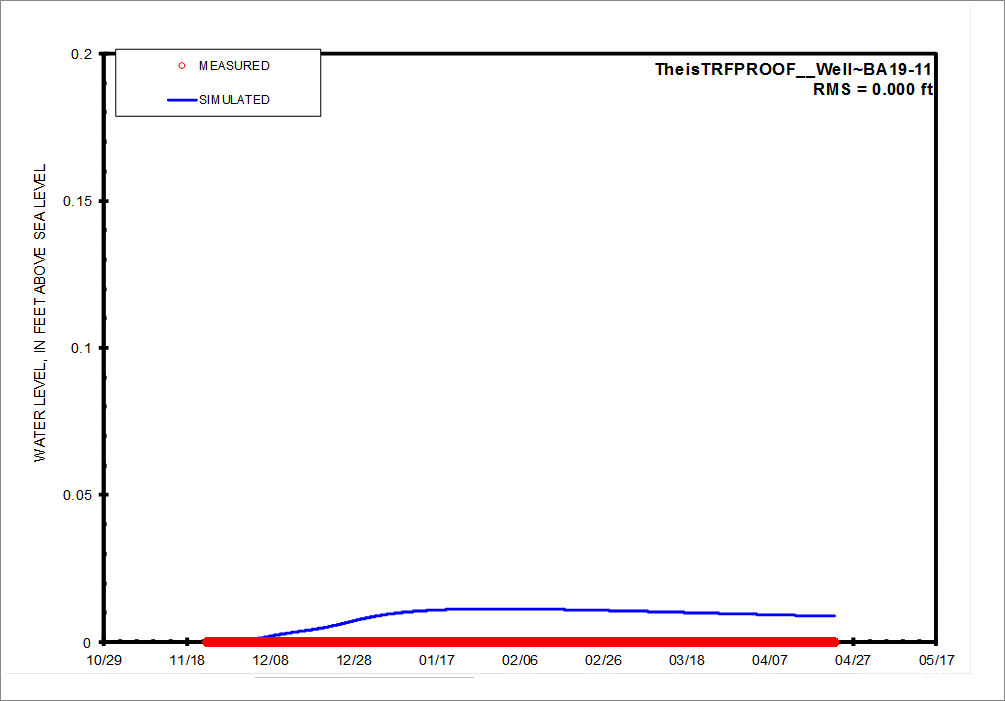

Supplement: Supplementary file 2 [file gwat0051-0322-SD2.zip › HypotheticalModel/MF/Hydros/HypoModel__Well~BA19-11.gif]

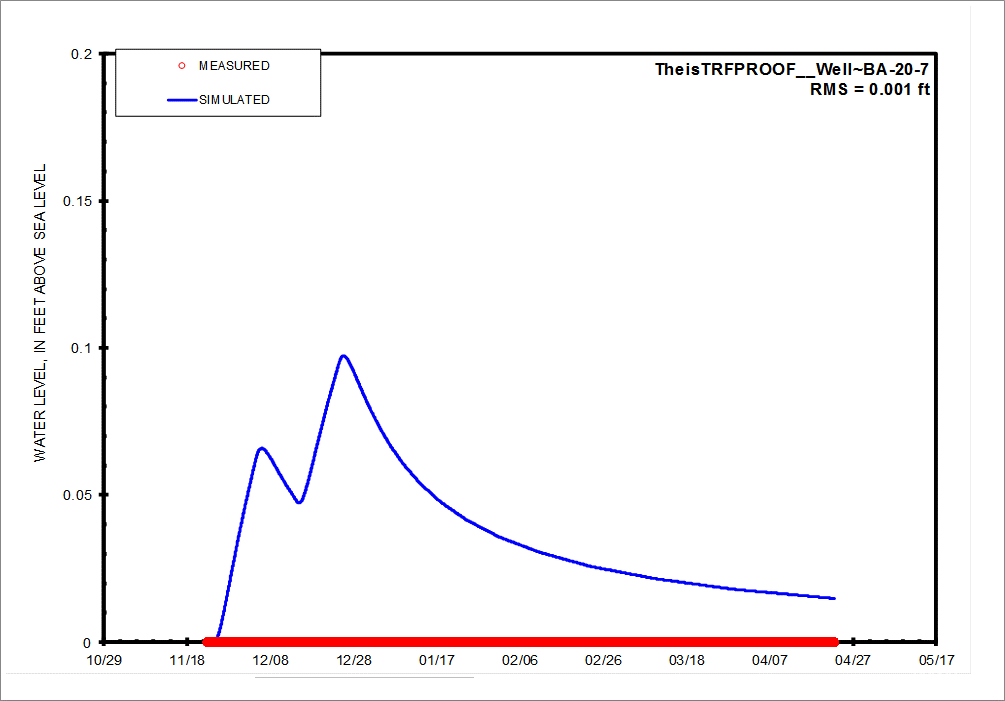

Supplement: Supplementary file 2 [file gwat0051-0322-SD2.zip › HypotheticalModel/MF/Hydros/HypoModel__Well~BA-20-7.gif]

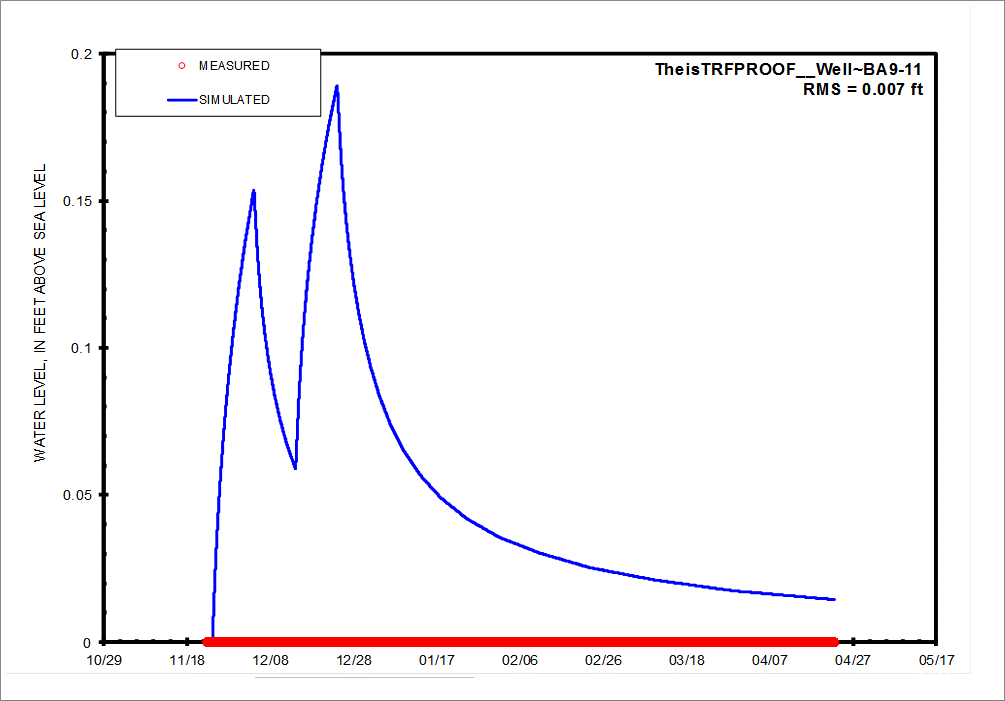

Supplement: Supplementary file 2 [file gwat0051-0322-SD2.zip › HypotheticalModel/MF/Hydros/HypoModel__Well~BA9-11.gif]

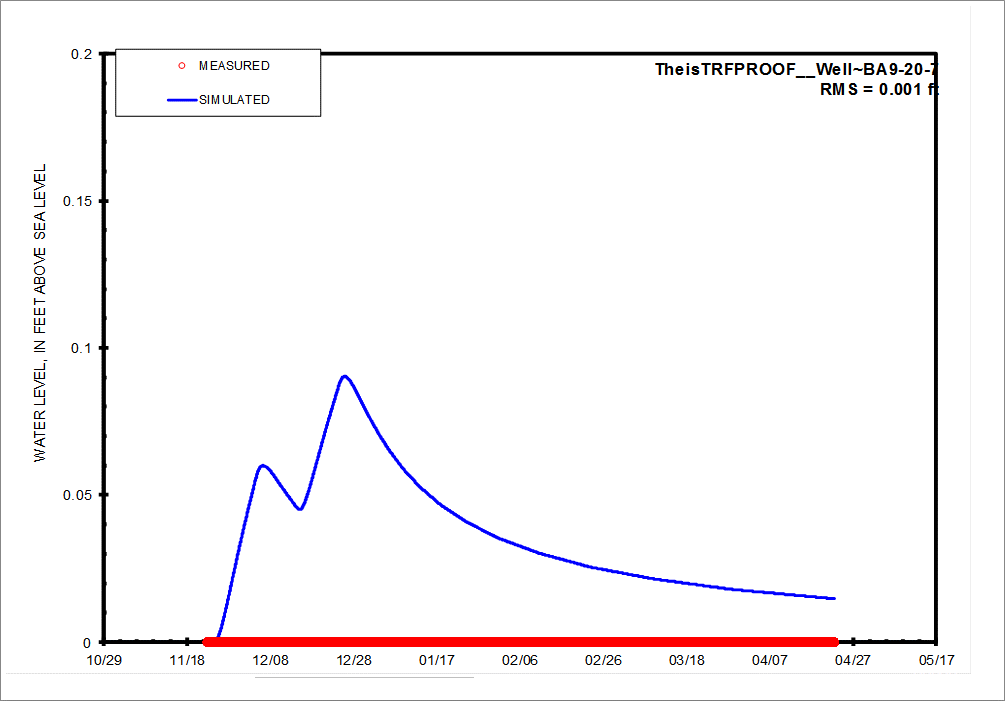

Supplement: Supplementary file 2 [file gwat0051-0322-SD2.zip › HypotheticalModel/MF/Hydros/HypoModel__Well~BA9-20-7.gif]

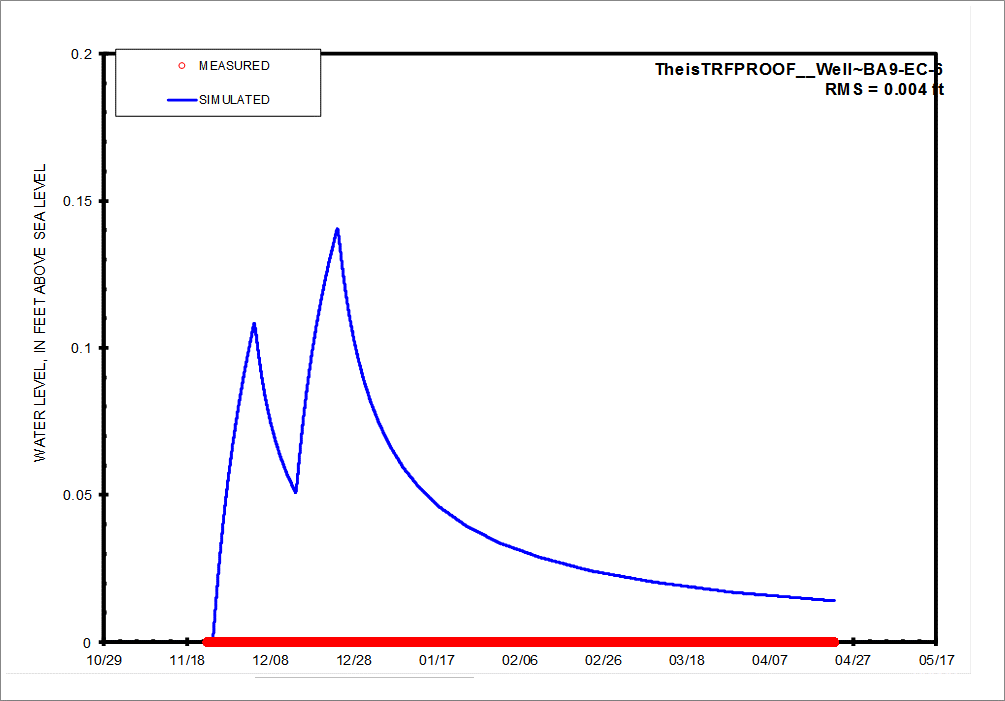

Supplement: Supplementary file 2 [file gwat0051-0322-SD2.zip › HypotheticalModel/MF/Hydros/HypoModel__Well~BA9-EC-6.gif]

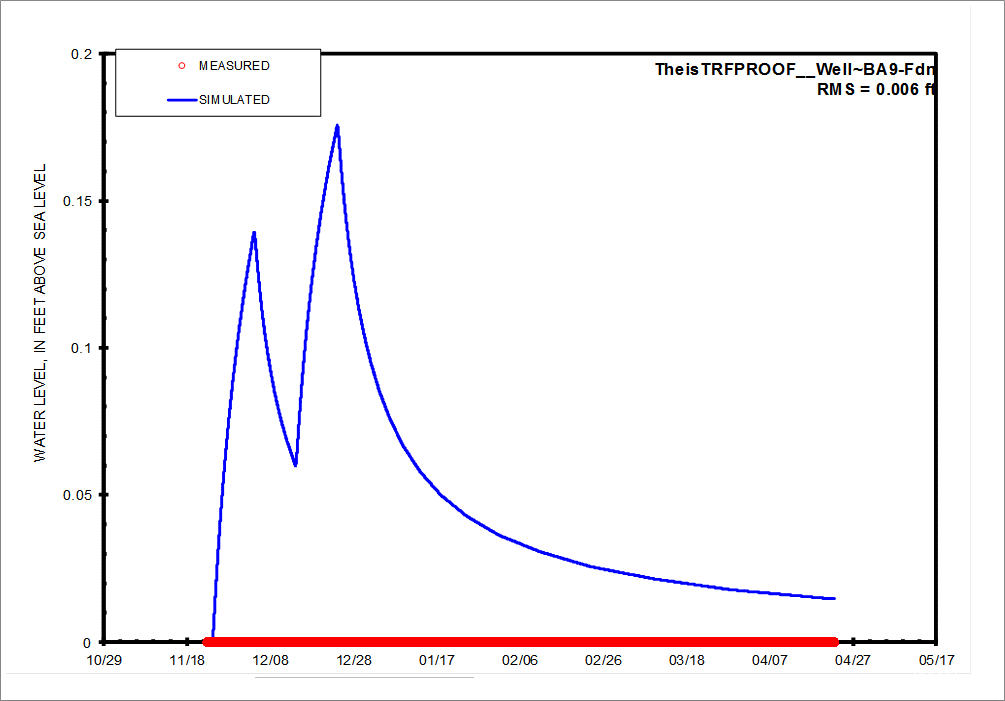

Supplement: Supplementary file 2 [file gwat0051-0322-SD2.zip › HypotheticalModel/MF/Hydros/HypoModel__Well~BA9-Fdn.gif]

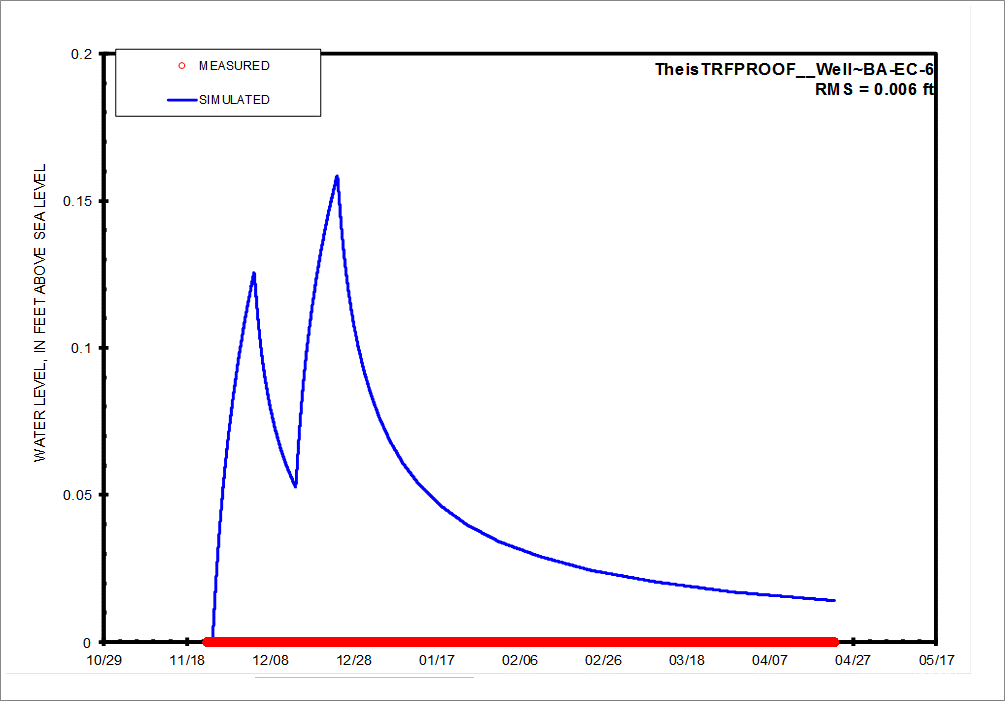

Supplement: Supplementary file 2 [file gwat0051-0322-SD2.zip › HypotheticalModel/MF/Hydros/HypoModel__Well~BA-EC-6.gif]

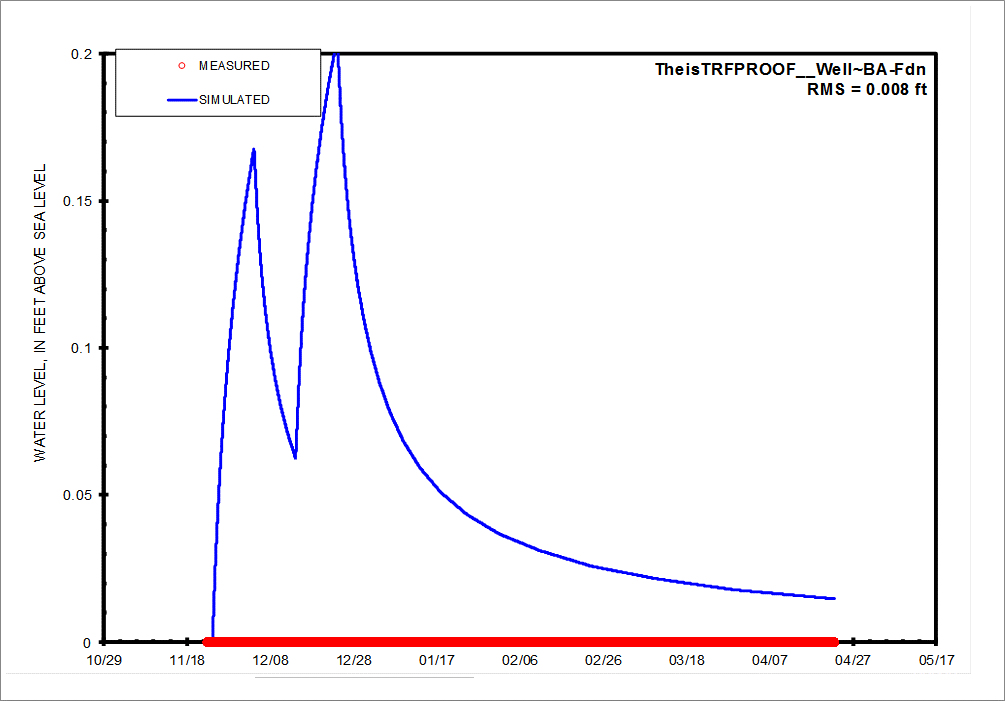

Supplement: Supplementary file 2 [file gwat0051-0322-SD2.zip › HypotheticalModel/MF/Hydros/HypoModel__Well~BA-Fdn.gif]

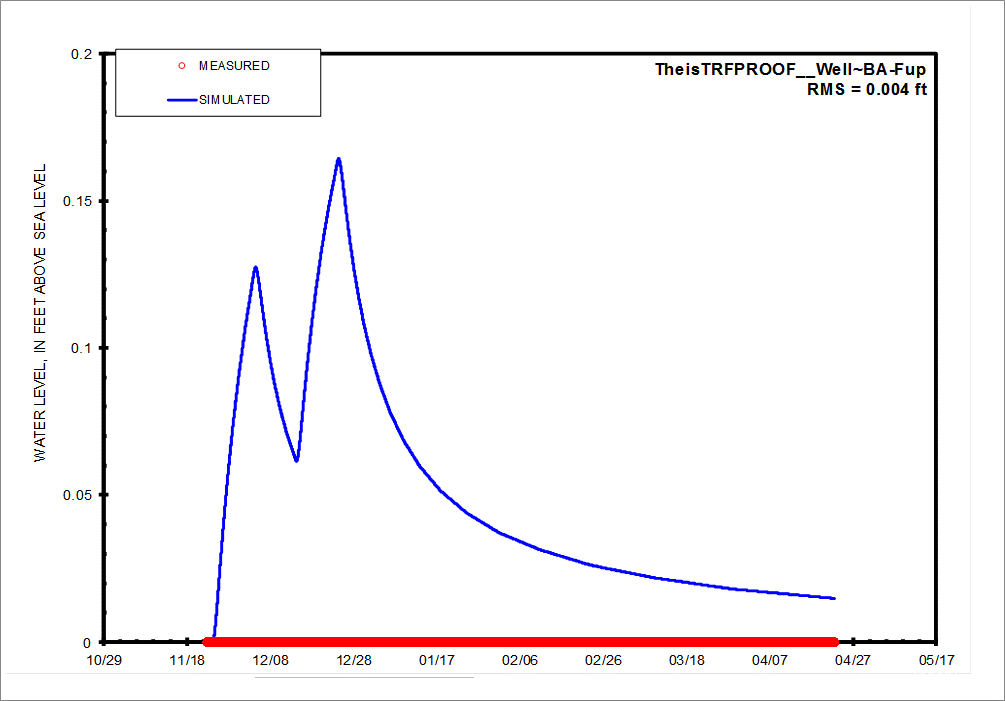

Supplement: Supplementary file 2 [file gwat0051-0322-SD2.zip › HypotheticalModel/MF/Hydros/HypoModel__Well~BA-Fup.gif]

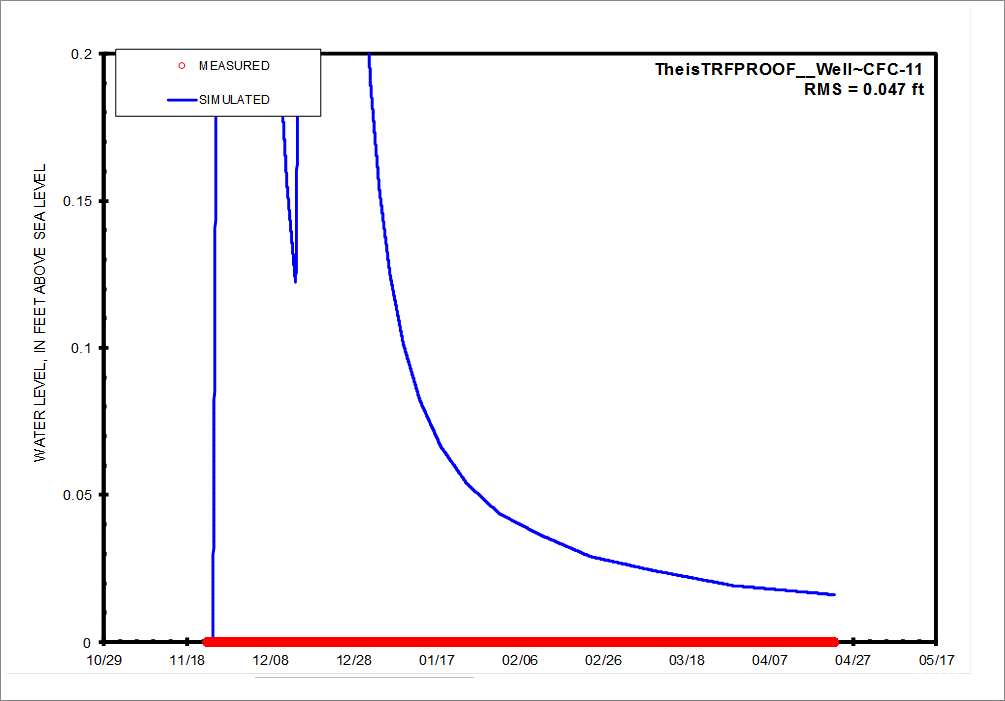

Supplement: Supplementary file 2 [file gwat0051-0322-SD2.zip › HypotheticalModel/MF/Hydros/HypoModel__Well~CFC-11.gif]

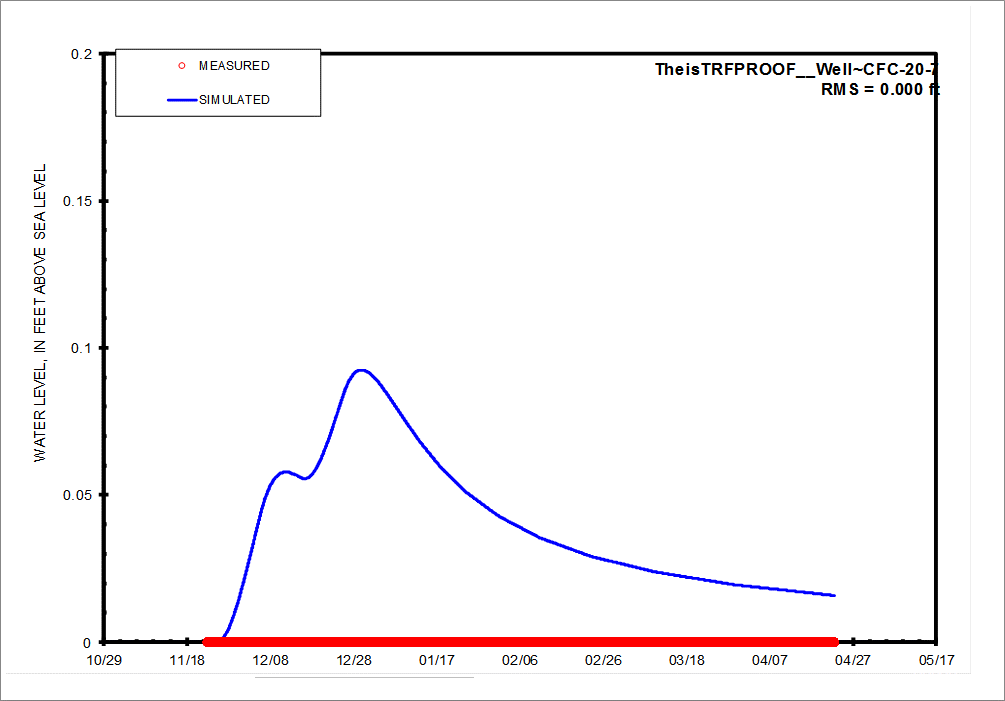

Supplement: Supplementary file 2 [file gwat0051-0322-SD2.zip › HypotheticalModel/MF/Hydros/HypoModel__Well~CFC-20-7.gif]

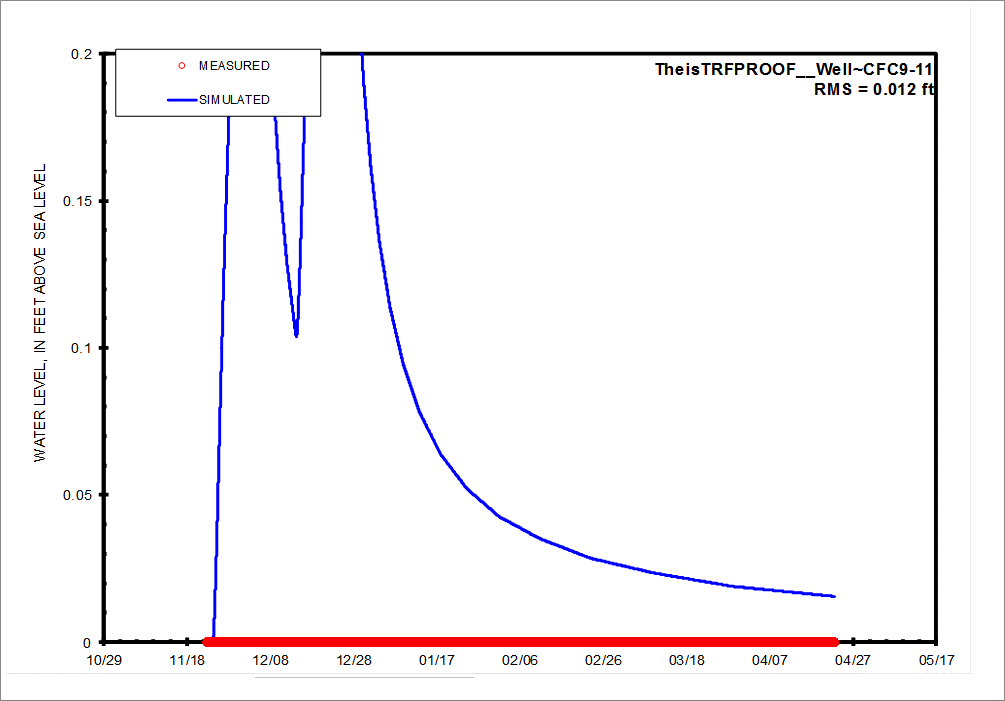

Supplement: Supplementary file 2 [file gwat0051-0322-SD2.zip › HypotheticalModel/MF/Hydros/HypoModel__Well~CFC9-11.gif]

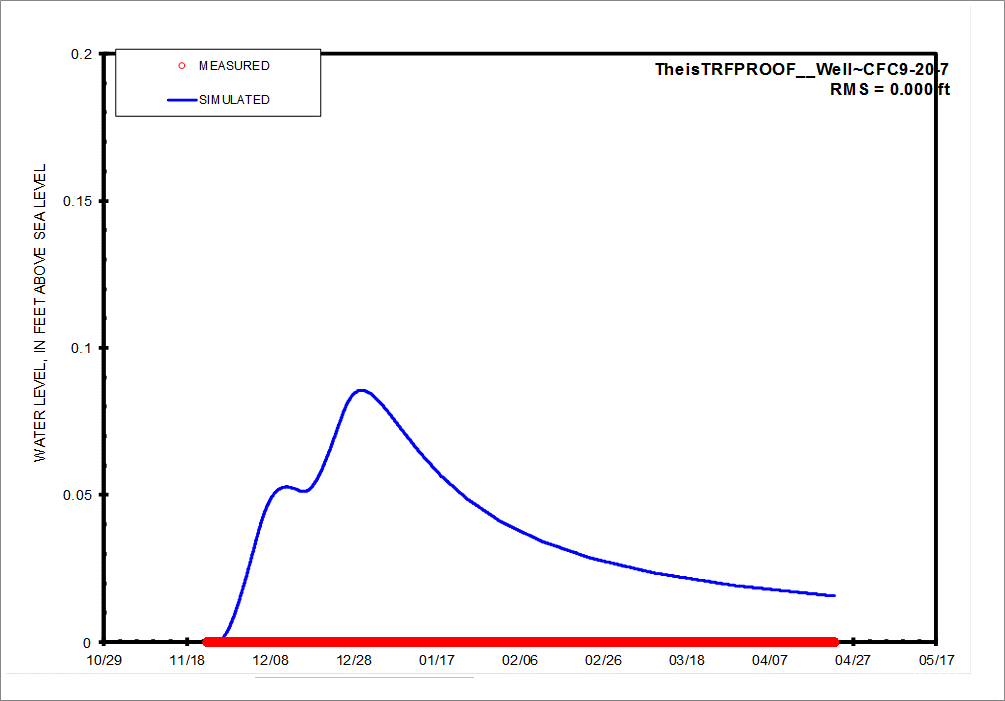

Supplement: Supplementary file 2 [file gwat0051-0322-SD2.zip › HypotheticalModel/MF/Hydros/HypoModel__Well~CFC9-20-7.gif]

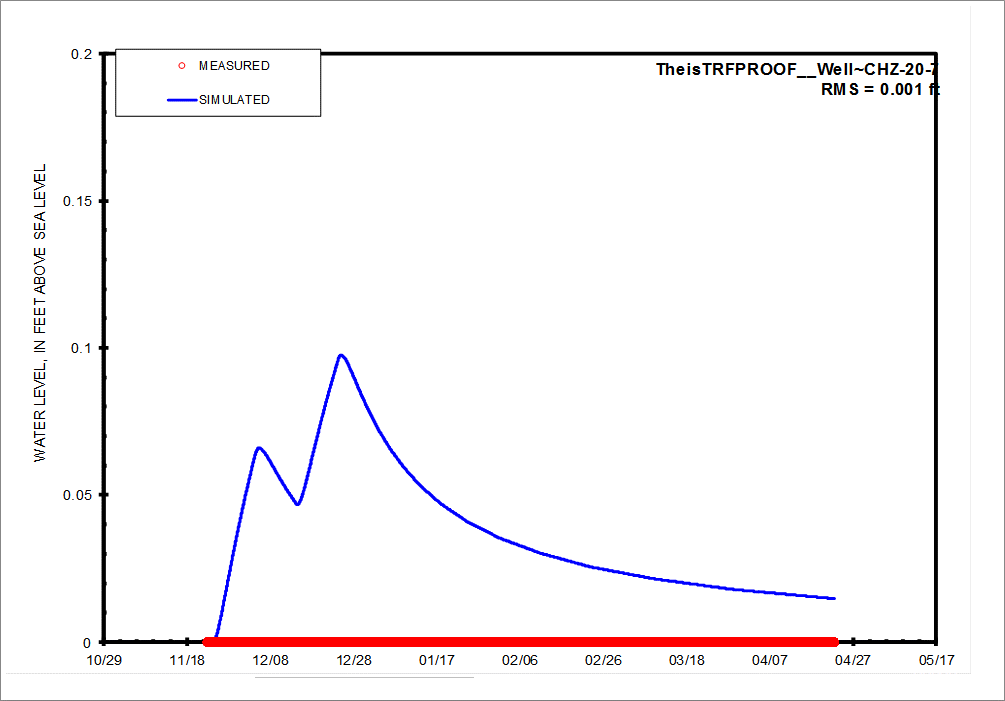

Supplement: Supplementary file 2 [file gwat0051-0322-SD2.zip › HypotheticalModel/MF/Hydros/HypoModel__Well~CHZ-20-7.gif]

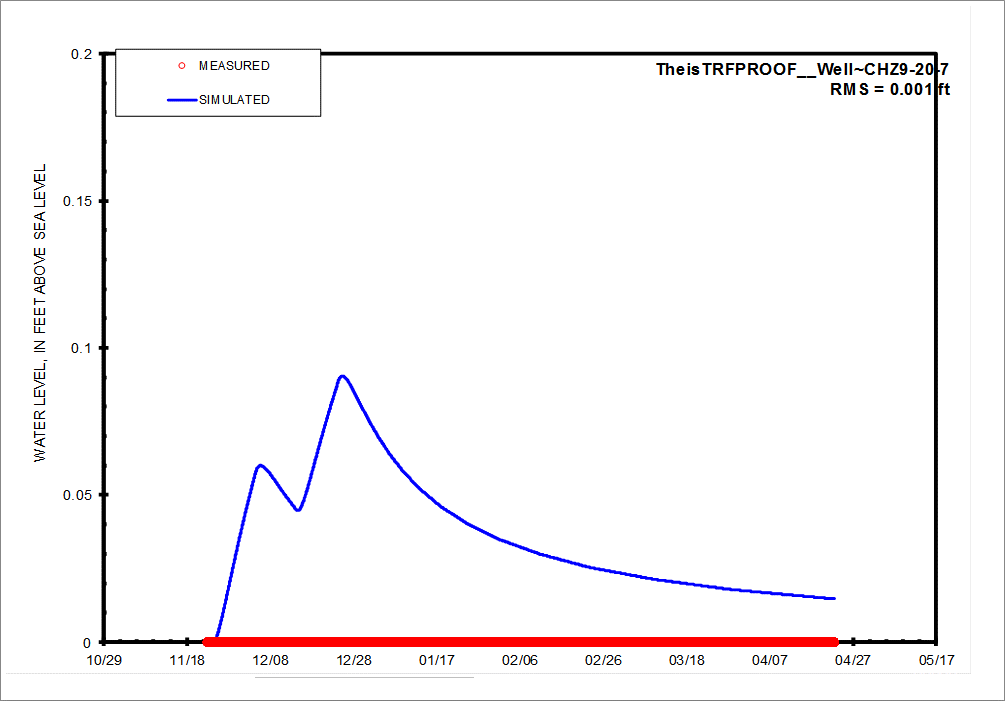

Supplement: Supplementary file 2 [file gwat0051-0322-SD2.zip › HypotheticalModel/MF/Hydros/HypoModel__Well~CHZ9-20-7.gif]

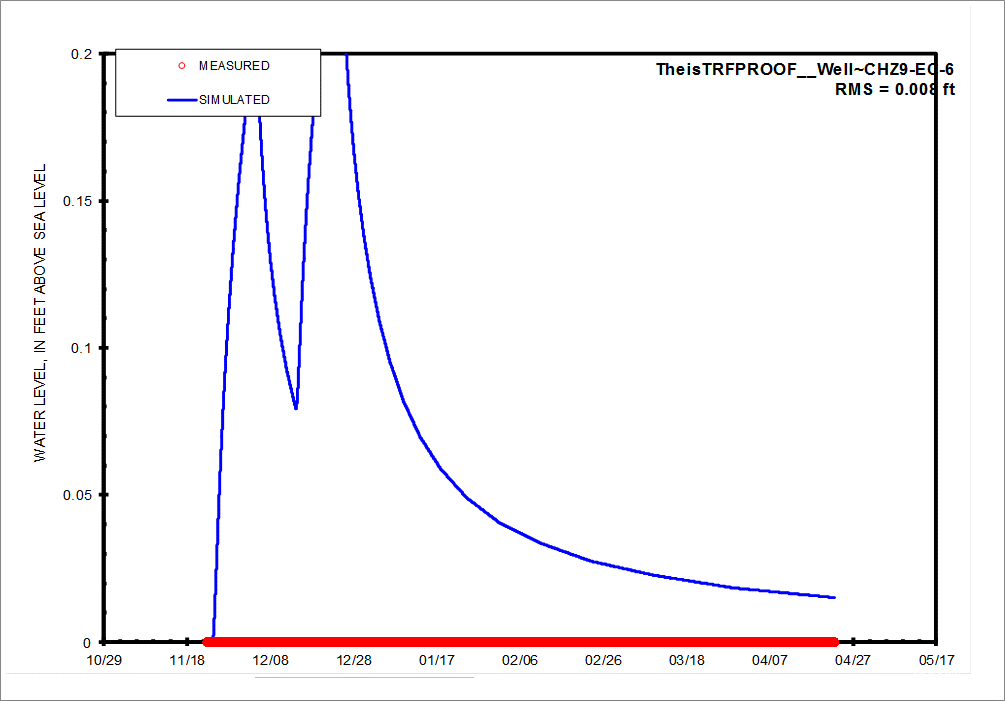

Supplement: Supplementary file 2 [file gwat0051-0322-SD2.zip › HypotheticalModel/MF/Hydros/HypoModel__Well~CHZ9-EC-6.gif]

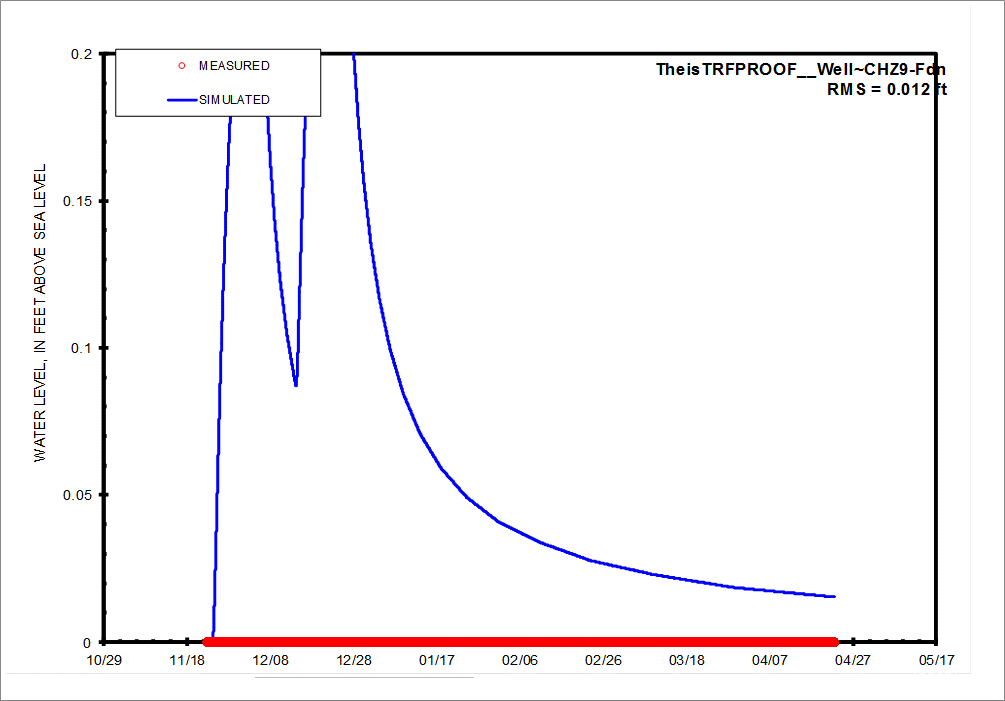

Supplement: Supplementary file 2 [file gwat0051-0322-SD2.zip › HypotheticalModel/MF/Hydros/HypoModel__Well~CHZ9-Fdn.gif]

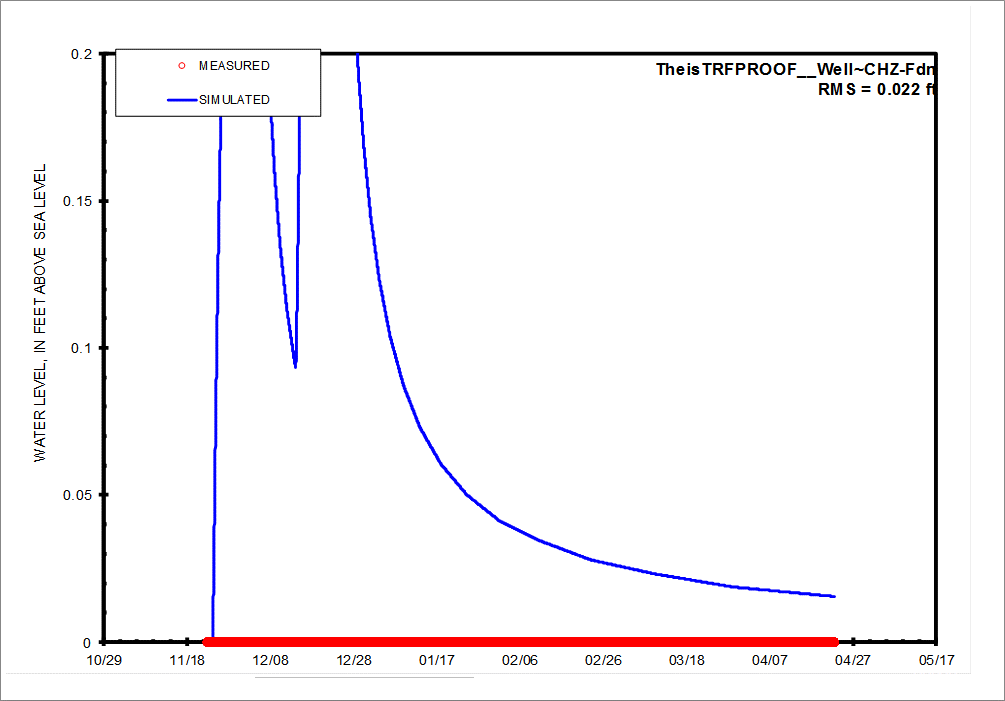

Supplement: Supplementary file 2 [file gwat0051-0322-SD2.zip › HypotheticalModel/MF/Hydros/HypoModel__Well~CHZ-Fdn.gif]

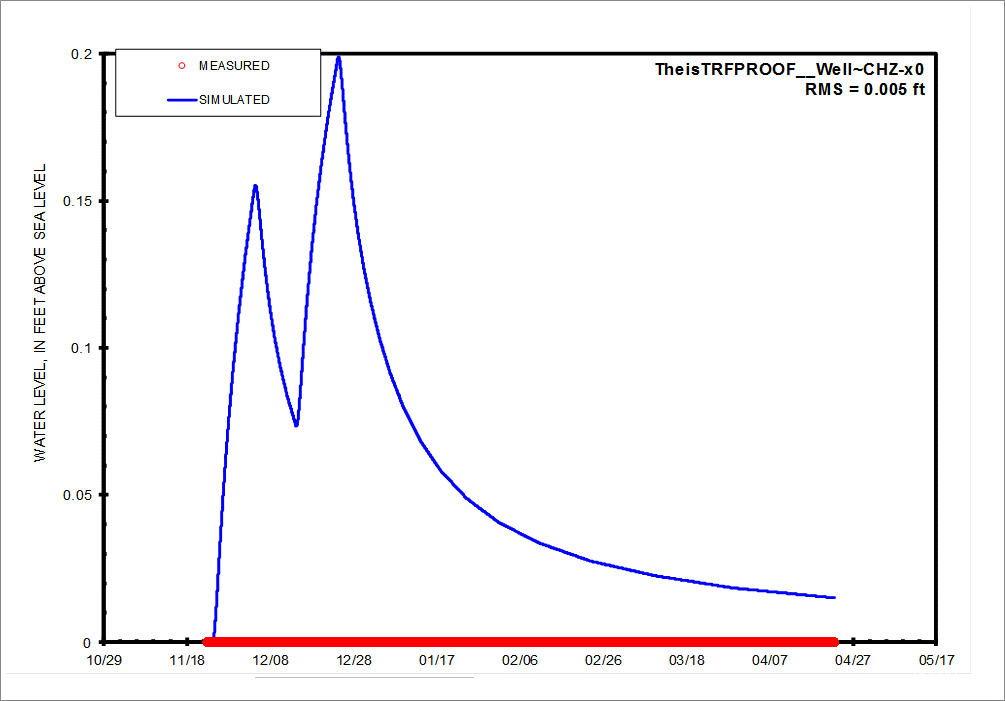

Supplement: Supplementary file 2 [file gwat0051-0322-SD2.zip › HypotheticalModel/MF/Hydros/HypoModel__Well~CHZ-x0.gif]

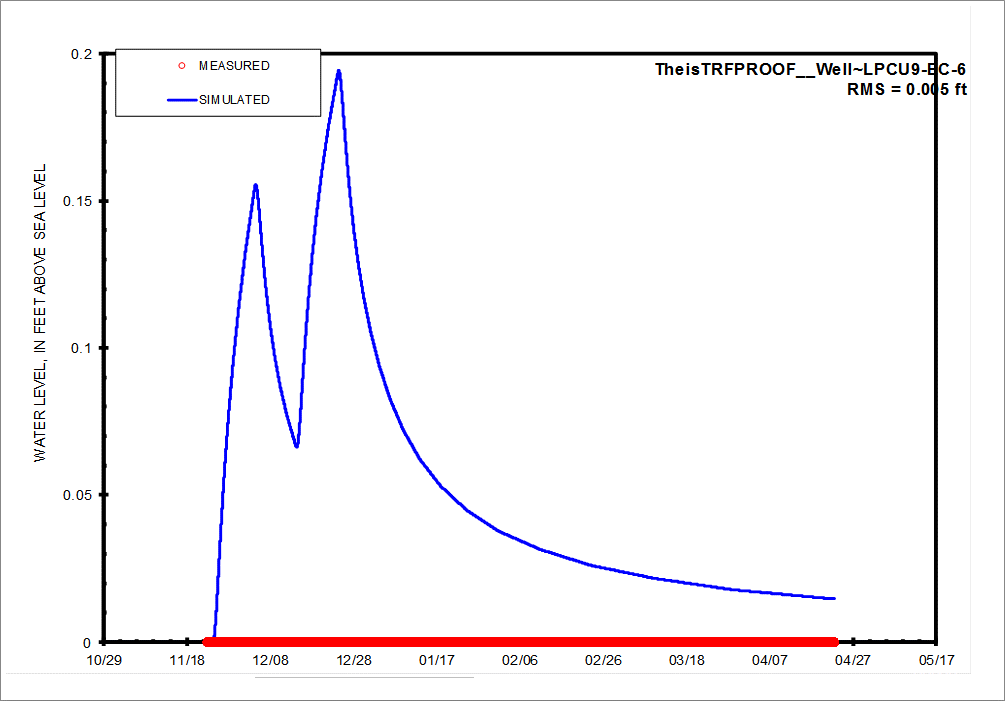

Supplement: Supplementary file 2 [file gwat0051-0322-SD2.zip › HypotheticalModel/MF/Hydros/HypoModel__Well~LPCU9-EC-6.gif]

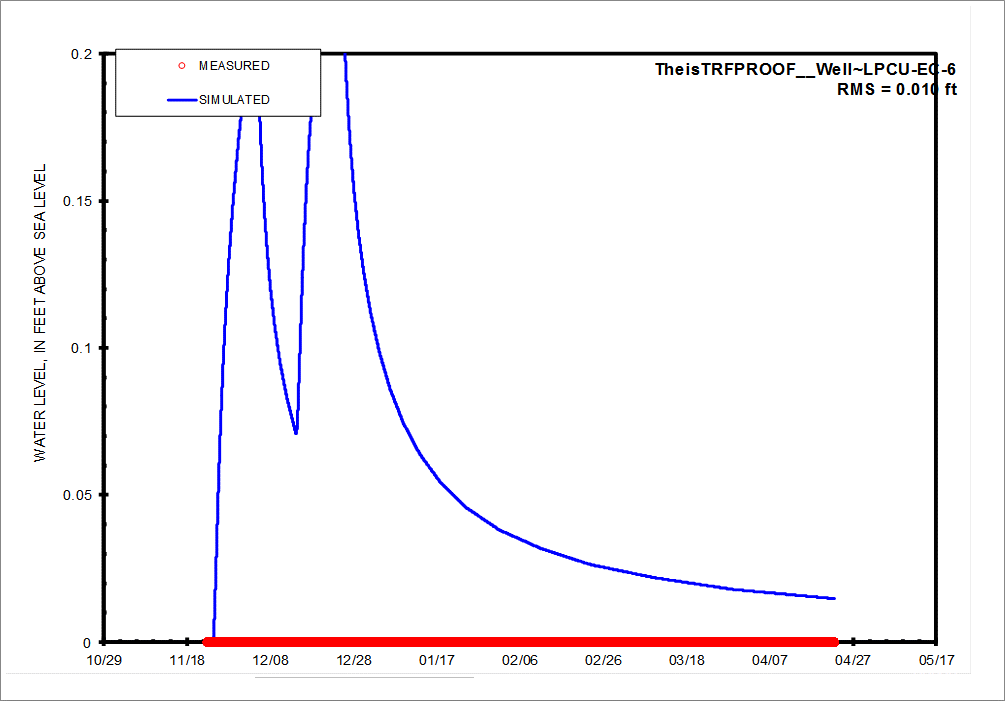

Supplement: Supplementary file 2 [file gwat0051-0322-SD2.zip › HypotheticalModel/MF/Hydros/HypoModel__Well~LPCU-EC-6.gif]

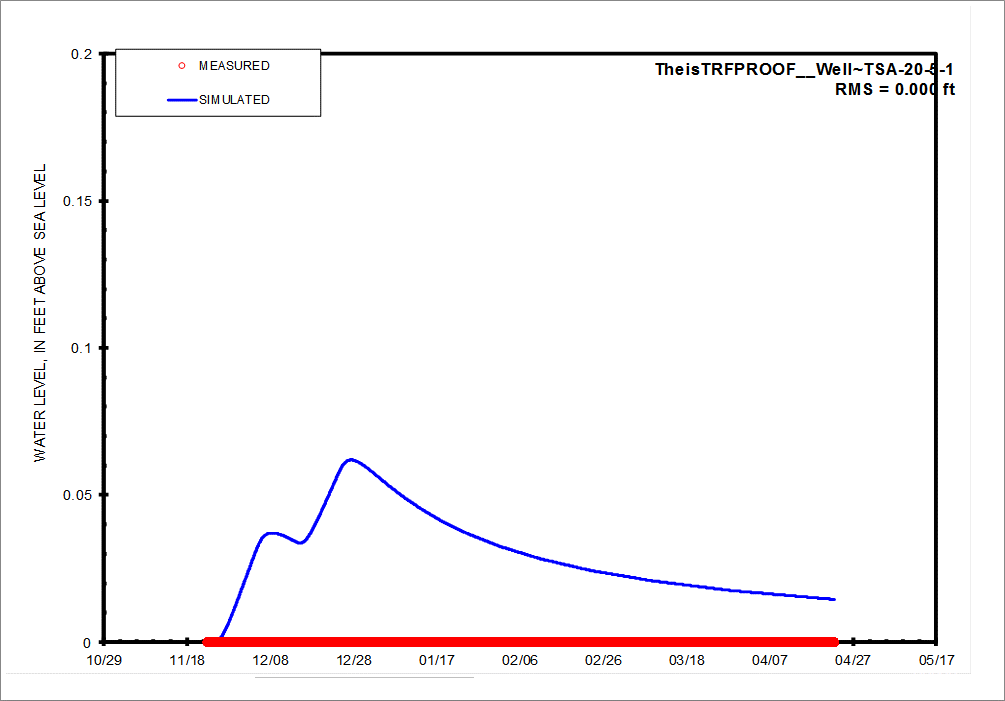

Supplement: Supplementary file 2 [file gwat0051-0322-SD2.zip › HypotheticalModel/MF/Hydros/HypoModel__Well~TSA-20-5-1.gif]

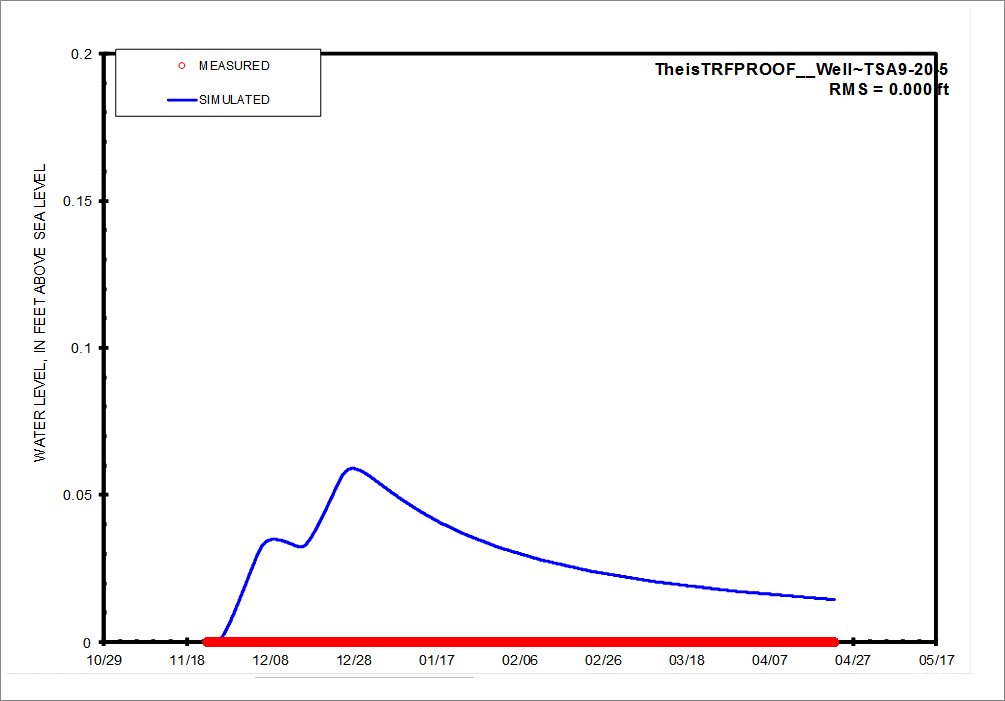

Supplement: Supplementary file 2 [file gwat0051-0322-SD2.zip › HypotheticalModel/MF/Hydros/HypoModel__Well~TSA9-20-5.gif]
